# Synthetic protein-conductive membrane nanopores built with DNA

**DOI:** 10.1038/s41467-019-12639-y

**Published:** 2019-11-04

**Authors:** Tim Diederichs, Genevieve Pugh, Adam Dorey, Yongzheng Xing, Jonathan R. Burns, Quoc Hung Nguyen, Marc Tornow, Robert Tampé, Stefan Howorka

**Affiliations:** 1 Institute of Biochemistry, Biocenter, Goethe University Frankfurt, Max-von-Laue Str.9, 60438 Frankfurt/M., Germany; 2 Department of Chemistry, Institute of Structural Molecular Biology, University College London, London, WC1H 0AJ UK; 3 Molecular Electronics, Technical University of Munich, Theresienstraße 90, 80333 Munich, Germany; 4 Fraunhofer Research Institution for Microsystems and Solid State Technologies (EMFT), Hansastraße 27d, 80686 Munich, Germany; 5 Center of Nanoscience (CeNS), Ludwig-Maximilians-University, Schellingstraße 4, 80799 Munich, Germany

**Keywords:** Biophysical chemistry, DNA nanostructures

## Abstract

Nanopores are key in portable sequencing and research given their ability to transport elongated DNA or small bioactive molecules through narrow transmembrane channels. Transport of folded proteins could lead to similar scientific and technological benefits. Yet this has not been realised due to the shortage of wide and structurally defined natural pores. Here we report that a synthetic nanopore designed via DNA nanotechnology can accommodate folded proteins. Transport of fluorescent proteins through single pores is kinetically analysed using massively parallel optical readout with transparent silicon-on-insulator cavity chips vs. electrical recordings to reveal an at least 20-fold higher speed for the electrically driven movement. Pores nevertheless allow a high diffusive flux of more than 66 molecules per second that can also be directed beyond equillibria. The pores may be exploited to sense diagnostically relevant proteins with portable analysis technology, to create molecular gates for drug delivery, or to build synthetic cells.

## Abstract (web summary)

Nanopores have a wide range of applications in the field of sensing. Here the authors report on synthetic nanopores made of DNA and designed for the transit of folded proteins across membranes to allow for biosensing.

## Introduction

Membrane nanopores are relevant for science and technology^1–8^. The biological function of shuttling molecular cargo across cell membranes^9–11^ can be exploited by engineering pores for enhanced cellular export of valuable molecules^12^. A more transformative approach is to use pores beyond their traditional biological role, such as for next-generation portable DNA sequencing and biosensing. In this analytical method, individual molecules pass through single membrane-embedded nanopores to cause detectable changes in the ionic pore current^3–5,7,8^. Narrow protein pores are highly suitable for sequencing because their 1–2 nm wide channels match the dimension of individual elongated translocating DNA strands^2,13–15^. Engineered protein pores are also versatile tools for studying the chemistry and biophysics of single molecules^16^, or to control flux of small bioactive compounds in and out of cells^6,17,18^.

Wider pores capable of transporting folded proteins or related cargo could realise similar benefits. For example, 5–10 nm wide pores could extend the analyte range for point-of-care diagnostics, environmental screening, or homeland security^7,19–21^. Wide and resealable pores could also function as molecular gates to release therapeutic proteins from drug-delivery vesicles. However, biological pores are not suitable for these applications^22^. They are either wide yet heterogeneous in diameter^23^, structurally defined but not sufficiently wide for protein transport^24,25^, or structurally complex^26,27^. De novo protein design^22^ of matching pores is currently too challenging, even though it is possible to obtain channels by arranging membrane-spanning peptides via covalently attached oligonucleotide scaffolds that are outside the membrane^28,29^.

Synthetic nanopores solely composed of DNA are an attractive alternative towards a wider lumen given the ease of rationally designing defined nanoscale architectures with DNA nanotechnology^30–34^. Indeed, DNA NPs carrying membrane anchors^35–40^ have been constructed using a basic design of six hexagonally arranged DNA duplexes that enclose a 2-nm-wide hollow channel^35–39^, along with wider versions^41,42^ which facilitate flux of double-stranded DNA^42^. While the synthesis of wide DNA nanopores is one crucial step, it is equally important to provide evidence of protein transport. The scientifically desirable analysis of single pores may be achieved with classical electrical recordings, yet transport can only be indirectly inferred from current fluctuations in low-throughput fashion. A better option is to measure diffusion flux in a massively parallel format with optical-readout silicon-on-insulator (SOI) chips to yield statistically relevant insights. Nevertheless, a synergistic combination of both methods would be ideal to better understand how electrophoretic vs. diffusion influence fundamental variables, such as transport speed.

Here we report on a DNA nanopore capable of transporting folded proteins as determined with electrical measurements and high-throughput diffusion flux analysis. The rationally designed DNA pores are assembled to yield the expected defined dimensions. Transport through the membrane-spanning channel is confirmed for two differently sized proteins, and translocation speed is at least 20-fold faster under electrophoresis than for diffusion-driven mode. Our study helps to better understand movement through nanoscale confined space, overcomes several limitations of biogenic and synthetic membrane nanopores, and opens up applications across biosensing and synthetic biology.

## Results

### Pore design

**Fig. 1:**
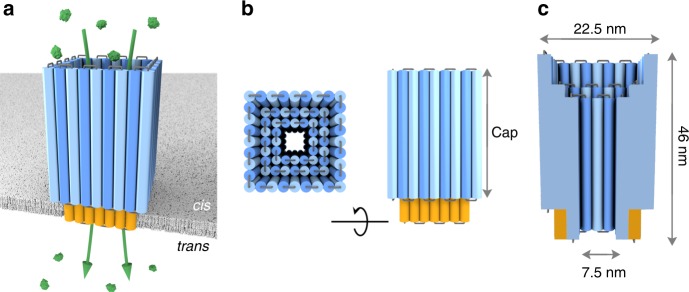
The rationally designed large membrane-spanning DNA nanopore NP. **a** The pore is composed of squarely arranged DNA duplexes, which are illustrated as blue and orange cylinders. The latter carry cholesterol lipid anchors for membrane insertion. Protein trypsin (green) can pass via the pore from the *cis* to the *trans* side of the membrane. **b** Top-down and side views of the nanopore. **c** Cross-sectional side view illustrating the geometry of the pore lumen with annotated dimensions

The membrane-spanning DNA nanopore NP was designed with the caDNAno software^43^ and is composed of parallel aligned DNA duplexes assembled in square lattice fashion (Fig. 1a, b, Supplementary Fig. 1). The duplexes are interlinked by cross-overs (Fig. 1a, grey loops; Supplementary Fig. 1)^30,31^. The pore features a cap region (blue) and a membrane-spanning region (orange, Fig. 1a, b). Its total height is 46 nm and the external maximum width measures 22.5 nm (Fig. 1b, c).

In NP’s cap region of 35 nm height, the pore wall is composed of up to three duplex layers to increase structural stability (Fig. 1b, c). In the membrane-spanning part, the wall is two-duplexes thick to decrease the overall pore-spanning area for facile membrane insertion (Fig. 1a, c). The transmembrane section carries a total of 24 lipid anchors composed of cholesterol to facilitate membrane insertion (Supplementary Fig. 1). By placing the anchors in a recessed pore environment (Fig. 1b), the formation of hydrophobically clustered pore oligomers can be suppressed. The lumen of the pore has a cross-sectional area of 7.5 × 7.5 nm^2^ and features a wider opening at its top to facilitate the entrance of biomolecules. In the membrane-inserted state, the pore is expected to enable transport across the membrane for protein cargo (green) smaller than the pore’s channel width (Fig. 1a).

### Pore assembly

**Fig. 2:**
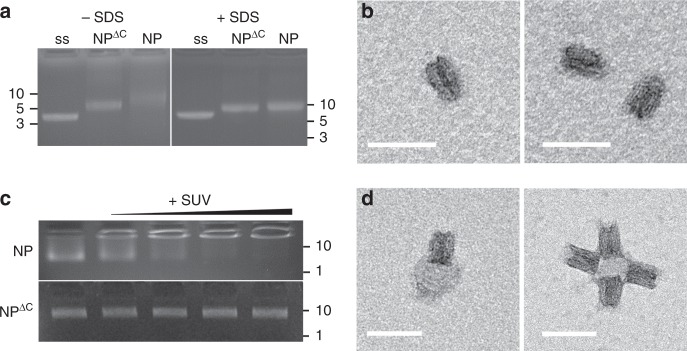
Assembly, purity, dimensions, and membrane-interaction of DNA nanopores NP and NP^ΔC^. **a** Gel electrophoretic analysis of scaffold strand (ss), nanopores NP^ΔC^ and NP without and with detergent SDS, respectively. The position and kilo base pair length of the dsDNA markers are annotated at the sides of the electropherograms. **b** Representative transmission electron microscopy (TEM) images of negatively stained NP^ΔC^. Scale bar, 50 nm. **c** Gel electropherogram of NP and NP^ΔC^ incubated with no (leftmost lane) or increasing amounts of small unilamellar vesicles (SUVs) ranging in concentrations from 6.9 to 12.5 nM. The upshifted bands of lipid anchor-bearing NP indicate favourable interactions with bilayer membranes. The interaction does not occur for anchor-free NP^ΔC^. The position of the two dsDNA markers with a length of 10 and 1 kbp is given at the right of the gels. **d** Representative TEM images of negatively stained NP inserted into SUVs. Scale bar, 50 nm. Source data are provided as a Source Data file

Two types of DNA nanostructure were generated: a pore with cholesterol lipid anchors, NP, and one without cholesterol lipid anchors, termed NP^ΔC^. The NP^ΔC^ pore is assembled via the scaffold-and-staple approach, whereby staple oligonucleotides direct the folding path of a long single-stranded DNA scaffold^30,31^. The lipid anchor-free pore can then be converted into lipid-modified NP by decorating the transmembrane region with cholesterol-carrying oligonucleotides. The 2D DNA map and DNA sequences of component strands are shown in Supplementary Fig. 2 and Supplementary Dataset 1, respectively. Assembly of NP^ΔC^ was analysed via electrophoresis to yield a single defined band (Fig. 2a, panel −SDS), implying a homogeneous population of folded products. The pore band migrated at a different height than the scaffold strand (ss) (Fig. 2a), indicating complete assembly. Pore NP with cholesterol anchors also led to a defined band when analysed in detergent SDS (Fig. 2a, panel +SDS) to suppress streaking caused by hydrophobic interactions with the gel matrix or by pore aggregation (Fig. 2a, panel −SDS)^37^. The DNA origami pores with a molar mass of 4.87 MDa were purified via size-exclusion chromatography (Supplementary Fig. 3) from excess staple oligonucleotides and used for biophysical analysis.

### Structural characterisation of the pores

Transmission electron microscopy (TEM) was applied to determine the dimensions of NP^ΔC^. The negatively stained samples featured isolated rectangular DNA nanopores   (Fig. 2b) whose  parallel aligned DNA duplexes are consistent with the design, similar to the different pore wall thicknesses at the upper pore entrance (Supplementary Fig. 4). Analyses of over 25 pores established a height of 31.5 ± 2.1 nm (±SD) and a width of 20.5 ± 1.7 nm. The latter is in excellent agreement with the expected width of 22 nm, while the height is slightly shorter than the 35 nm of the cap region. The total pore height of 46 nm is not completely apparent since the single-duplex-thin transmembrane region were not intensely stained.

The anchoring of cholesterol-tagged NP into lipid bilayers was established using a gel shift assay. The band for the nanopore was upshifted and co-migrated with small unilamellar vesicles (SUVs) that were unable to enter the gel (Fig. 2c). Increasing amounts of SUVs led to a complete conversion to the upshifted DNA band (Fig. 2c), implying that all pores interacted with the lipid bilayer. By contrast, NP^ΔC^ without lipid anchors did not produce any gel shift (Fig. 2c) as cholesterol is needed for membrane insertion. Pores with half the number of cholesterol anchors resulted in incomplete gel shifts (Supplementary Fig. 5).

Pore insertion via its membrane-spanning region was confirmed with TEM analysis. NP nanopores were incubated with SUVs of an average diameter of 50 nm and negatively stained. The striking TEM images show single and multiple pores embedded into vesicles (Fig. 2d, Supplementary Fig. 6), thereby validating the concept of faciliated DNA pore insertion into curved membranes^37,44^. In addition, the narrower part of pore lumen pointed towards the vesicle membrane (Fig. 2d, Supplementary Fig. 6), consistent with the predicted orientation of the membrane-inserted DNA nanopore (Fig. 1c). In analogy to small DNA pores^44–46^, the mechanism and energetics for membrane insertion of the large pore most likely involves a first step that tethers the pore to the bilayer without puncturing, possibly in a non-perpendicular orientation to the membrane. In a second step, the pore reorients itself to span the lipid bilayer.

### Ionic transport through individual pores

**Fig. 3:**
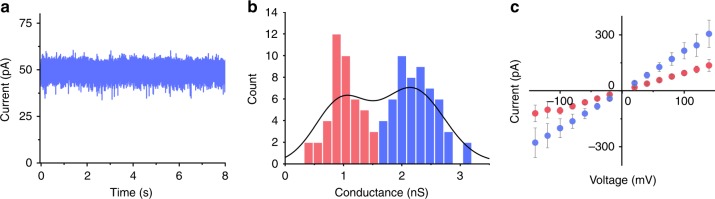
Electrical recordings confirm the membrane-spanning nature of DNA nanopore NP. **a** Representative ionic current trace of a single nanopore in 1 M KCl, 10 mM HEPES pH 8.0, and at +20 mV relative to the *cis* side of the membrane (Fig. 1a). **b** Histogram of channel conductances obtained from approximately 100 independent single-channel recordings at +20 mV to +50 mV. Red and blue indicate the low and high conductance states of pores, respectively. **c** Current–voltage (*IV*) curves displaying the averages and standard deviations from 10 independent single-channel current traces. The low and high conductance states of the pores are shown in blue and red, respectively. Electrical recordings were measured using the Orbit 16 device which is grounded at the* cis* chamber. Source data are provided as a Source Data file

The conductance properties of membrane-spanning NP nanopores were analysed with single-channel current recordings. Individual pores were inserted into a planar lipid bilayer and a potential was applied across the membrane to induce flow of electrolyte ions^10,39,47^. Under standard electrolyte conditions, a constant current of 49.5 pA was observed (Fig. 3a) at a potential of +20 mV relative to the *cis* side of the pore. The corresponding conductance distribution of 98 pores had one maximum at 2.37 ± 0.30 nS (*n* = 56, ±SD, *n* is the number of independent pore insertions) (Fig. 3b). In agreement with the wide pore lumen, the conductance is 2.6-fold higher in comparison to a reference DNA pore of 2 nm diameter^37^. The conductance is lower than the theoretical value of 6.7 nS calculated for the known pore geometry of NP (see Methods), but this is expected as simple calculations incorrectly assume a constant mobility of electrolyte ions in negatively charged nanopores^48^. The low conductance suggests that ionic leakiness found in simulations of DNA structures^49–51^ is not a major influence for this nanopore. Otherwise, a higher conductance would have been found. In the conductance histogram, a second peak was evident at 1.18 ± 0.42 nS (*n* = 43) (Fig. 3b), which suggests a smaller conductance population, likely induced by voltage. High-to-low conductance switches are often seen at voltages exceeding 80 mV (Supplementary Fig. 7). The voltage-dependent gating may be caused by partial unzipping of DNA strands^36,49^ and could be reduced by forming a more covalently closed nanostructure^52^. The voltage ramps established that the nanopore was of ohmic behaviour; apparently, the structural asymmetry of the pore does not influence voltage-dependent conductance (Fig. 3c).

### Protein transport through individual pores

**Fig. 4:**
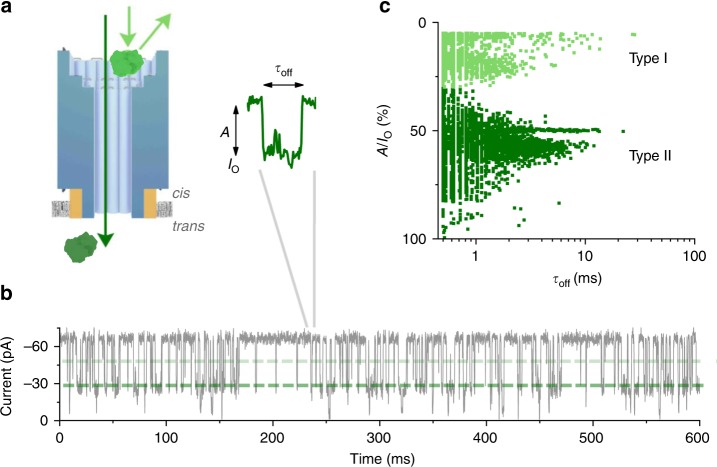
Transport of protein trypsin through DNA nanopore NP. **a** Scheme of the DNA nanopore and its interaction with protein molecules (green). **b** Single-channel current trace recorded at −50 mV in the presence of trypsin (66.7 µM) at the *cis* side leading to blockade. **c** Scatter plot showing dwell time *τ*_off_ and amplitude *A* for single-channel current recordings with trypsin (66.7 μM) in the *cis* chamber. Points cluster into type I (light green) and type II (dark green) events. Each point in the diagram represents an individual encounter event of protein with the DNA nanopore. The scatter plot comprises 7282 data points from three independent DNA nanopore insertions. Electrical recordings were measured using the Orbit mini device which is grounded at the trans chamber. Source data are provided as a Source Data file

The transport of protein along the channel lumen of NP (Fig. 4a) was examined with single-channel current recordings. As model protein, trypsin with a diameter of 4.1 × 3.2 × 2.0 nm^3^ and an isoelectric point, pI, of 10.1 was selected  along with an electrophysiological buffer with pH 8.0 to render trypsin net positively charged. Upon its addition to the *cis* side at a concentration of 66.7 μM, current blockade events occurred (Fig. 4b). Their frequency increased with the protein concentration (Supplementary Fig. 8). The events  were characterised with their duration, *τ*_off_, and amplitude, *A* (Fig. 4b, inset). When each event was plotted with its *τ*_off_ and *A* as separate points in a scatter diagram, two types of events became apparent (Fig. 4c). Type I clustered at a blockade of 13.3 ± 6.1% (±SEM) (*n* = 3) and type II at blockade of 57.2 ± 8.5% (*n* = 3). Both events had a similar *τ*_off_ distribution ranging from 0.5 to 20 ms with type I featuring an average *τ*_off_ of 1.04 ± 0.17 ms (*n* = 3) and type II at 1.31 ± 0.29 ms (*n* = 3). The τ_off_ values were obtained from the fit to the single exponential decay distributions. Type II events were more frequent, comprising 5276 out of 7282 points of the distribution.

In line with related current signatures through inorganic pores^19,53^, type I events are interpreted as protein bumping at the funnel opening or as temporary entrapment without pore translocation leading to partial pore blockage (Fig. 4a, bright green). By contrast, type II events are considered to represent trypsin translocation through the entire DNA pore lumen (Fig. 4a, dark green). The translocation  may involve intermittent interactions of the positively charged trypsin to the negatively charged pore wall. Type II blockade events are voltage-dependent, occurring only at negative membrane potentials (Supplementary Figs. 9 and 10). Type I events, however, were apparent at both positive and negative membrane potentials, which is consistent with their assumed origin as intermittent collisions with the DNA nanopore.

To test whether type II blockade events were due to trypsin translocation, another protein, green fluorescent protein (GFP), was tested. GFP is larger than trypsin resembling a cylinder of length 4.2 nm and average diameter of 3 nm, with an N-terminal α-helix that extends an extra ~1 nm. GFP is thus expected to block the DNA pore lumen to a greater extent than trypsin. In addition, GFP has a different pI of 5.8 and is net negatively charged at pH 8.0, but net positively charged at the acidic pH of 5.0. A negatively charged protein is not expected to interact with the negatively charged pore walls. Indeed, when using an electrophysiological buffer of pH 8.0, no GFP translocation events were detected (Supplementary Fig. 11a). It is thought that in the absence of electrostatic interaction with the pore walls, GFP passes through the DNA nanopore lumen too quickly to be detected by the amplifier. However, when the pH of the electrophysiological buffer was dropped to pH 5.0, distinct blockade events occured after the addition of 200 nM GFP to the *cis* side (Supplementary Fig. 11b). In concordance with trypsin translocation, GFP translocation also clustered into type I and type II events when each event was plotted with its duration *τ*_off_ and amplitude *A* as separate points in a scatter diagram (Supplementary Fig. 11c). Type II events were voltage-dependent, occurring only at negative membrane potentials (Supplementary Fig. 11c, e). Type I events occurred, however, at both positive and negative potentials (Supplementary Fig. 11c, e). In agreement with the larger size of GFP, a more extensive blockade of type II events was seen, with an average blockade of 72.6 ± 8.9% (±SEM) (*n* = 3) compared to 57.2 ± 8.5% (*n* = 3) for trypsin. The blockade levels of type I events remain fairly constant, with an average value of 6.9 ± 2.2% (*n* = 3) for GFP compared to 13.3 ± 6.1% (*n* = 3) for trypsin.

These results support our hypothesis that type I events are caused by protein bumping at the funnel opening, whereas type II events correspond to protein translocating through NP. The GFP translocation data also support the idea that protein translocation can only be resolved after a sufficient reduction in the speed of translocation, in our experiments caused by an increased electrostatic interaction between the translocating protein and the pore wall. In line with this model, the proteins’ translocation duration at around 1 ms is strikingly two orders of magnitude slower than for a 20-nm-wide solid-state pore of similar charge polarity^54,55^. The driving force for protein translocation is of more complex nature. Electrophoresis cannot account for the translocation as the force at negative potentials would drive the positively charged trypsin at pH 8.0 (and GFP at pH 5.0) out of the pore (Supplementary Fig. 12). Most likely, the details of molecular transport must also consider the non-simplistic electric fields in negatively charged DNA nanopores, as apparent in molecular dynamics simulations^41^.

### Massively parallel optical analysis of protein transport

**Fig. 5:**
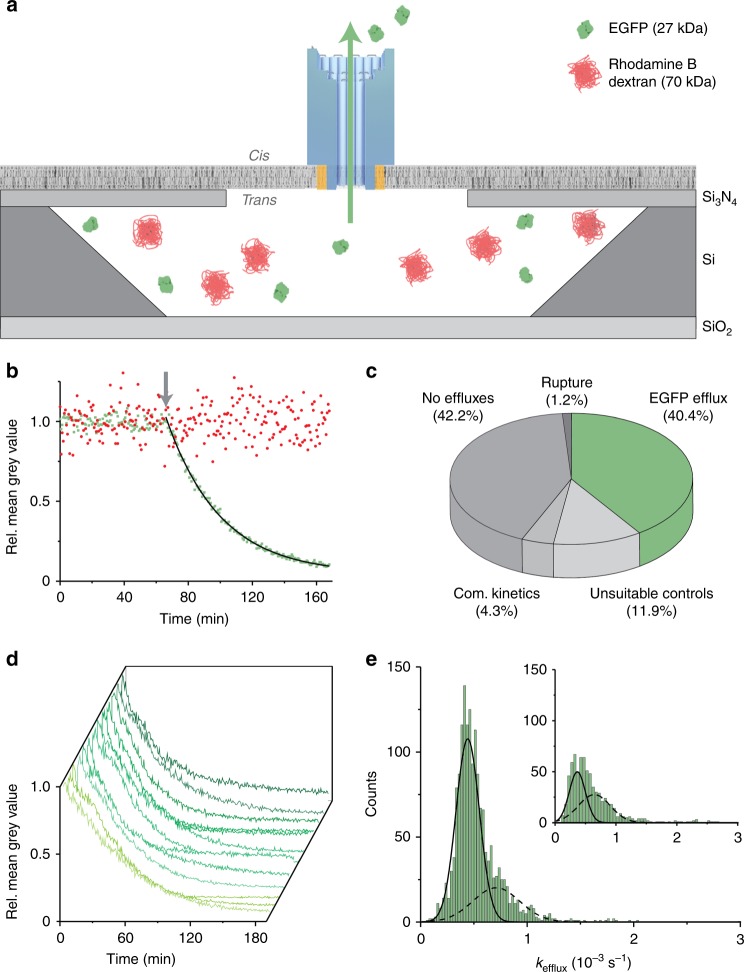
Kinetic analysis of protein transport through single DNA nanpores analysed using silicon chips. **a** Architecture of a transparent silicon-on-insulator (SOI) chip featuring a microcavity closed off by a membrane with  the embedded DNA nanopore NP. The microcavity contains a solution of enhanced green fluorescent protein (EGFP) (27 kDa) and Rhodamine B dextran (70 kDa) at the *trans* side and is separated by the solid-supported lipid bilayer (SLB) from the buffer reservoir (*cis* side). EGFP acts as flux analyte, whereas larger Rhodamine B dextran (70 kDa) is a negative control for pore transport  and indicates membrane rupture. The microcavity is not drawn to scale. **b** Exemplary normalised EGFP (green squares) and Rhodamine B dextran (red circles) fluorescence signals implying translocation of folded proteins through a DNA nanopore. The arrow (grey) shows the point of spontaneous membrane insertion of a nanopore. **c** The statistics of thousands of single translocation traces are classified in nanopore-mediated EGFP effluxes, membrane ruptures, complex kinetics, and inadequate controls as well as cavities without signal changes. The statistics were obtained  for 1 pM NP. **d** Multiple similar, normalised EGFP efflux traces from different SOI cavities indicate high structural homogeneity among nanopores. **e** DNA pore-mediated efflux traces were fitted monoexponentially with rate constant  *k*_efflux_ and the values are summarised in the histograms. The distribution of efflux constants was Gaussian fitted to reveal single nanopore efflux constants (*k*_eff single_) (solid line) and double nanopore efflux constants (*k*_efflux double_) (dashed line). The proportion of single nanopore efflux constants was larger for 1 pM DNA nanopore NP compared to 10 pM (inset). The total number of individual traces in the histograms are  1649 and 737, respectively. Source data are provided as a Source Data file

Recently, silicon chips gained attention for the optical characterisation of membrane protein kinetics, due to their high parallelism and throughput^56–60^. Therefore, protein translocation through individual NP DNA nanopores was analysed in the absence of electrical fields via an optical readout SOI chip^58^. The latter features 14,400 identical cavities with 50-fL volumes (*trans* side) connected by single solid-state nano-orifices of 80 nm diameter to a buffer reservoir (*cis* side). In the optical assay, fluorescent probes are encapsulated inside the chips’ arrayed cavities with a lipid bilayer spread across the cavities’ top (Fig. 5a). The stochastic insertion of one DNA nanopore per cavity leads to the diffusive outflow of fluorescent probes, which can be tracked by time-lapse fluorescence images of the cavity array. As a result, hundreds up to thousands of translocation processes can be visualised in parallel through individual nanopores.

The transport kinetics through single DNA nanopores were examined with enhanced green fluorescent protein (EGFP). The barrel-shaped protein of 27 kDa has a height of 4.2 nm and a diameter of 3.0 nm^61^, and is expected to translocate through the pore lumen of 7.5 × 7.5 nm^2^ cross-sectional area. By contrast, control probe Rhodamine B dextran (70 kDa, 8 nm hydrodynamic diameter^62^) should not pass the pore. In the experiments, EGFP (5 µM) and control probe Rhodamine B dextran (8 µM) were first sealed inside the cavities at efficiencies up to 81%, as determined by colocalisation of both probes (Supplementary Fig. 13). Addition of DNA nanopores and successive time-lapse recordings of the cavity array revealed exponentially decaying fluorescence signals for EGFP   reflecting pore-mediated protein efflux (Fig. 5b, green fluorescence).  In support of size-selective transport, the signal for negative control Rhodamine B dextran remained constant (Fig. 5b, red fluorescence). Statistical analysis of more than a thousand traces indicated high experimental quality because monoexponential EGFP decay was found for 40.4% of all sealed cavities (Fig. 5c). In contrast, only 1.2% of  the cavities showed fast declines for both fluorescence signals, and 42.2% had no signal changes implying high membrane stability (Fig. 5c, Supplementary Fig. 13). The remaining cavities featured complex kinetics such as increase in EGFP signals, or unsuitable controls, which have no, weak, or unexpected signals for Rhodamine B dextran. Further analyses of pore-mediated effluxes were carried out exclusively with traces showing monoexponential decays in the EGFP signal recordings and constant fluorescence for Rhodamine B dextran (Fig. 5d).

Efflux traces were analysed to determine the exponential rate constant *k*_efflux_ for protein translocation through single DNA nanopores. At a pore concentration of 3.2 nM, a heterogeneous distribution of *k*_efflux_ was obtained, indicating undesirable multiple nanopore insertions per cavity (Supplementary Fig. 14). By contrast, a considerably lower concentration of 10 pM yielded a total of 737 traces with two Gaussian-distributed *k*_efflux_ peaks (Fig. 5e, inset). The first peak at 3.66 ± 1.33 × 10^–4^ s^–1^ (±SD from the Gaussian fit) represents single-pore translocation, whereas the second peak of approximately twice the value at 6.44 ± 2.60 × 10^–4 ^s^–1^ stems from two DNA nanopores  simultaneously inserted in the membrane of an individual cavity. Further support for single-pore protein translocation was obtained with an even lower DNA pore concentration of 1 pM to diminish the proportion of cavities with two insertions. Indeed, *k*_efflux_ rate constants from more than 1600 traces (Fig. 5e) were more concentrated in the first peak of 4.42 ± 1.10 × 10^–4^ s^–1^. The rate constant implies that up to 66 individual EGFP molecules translocate through the pore per second. When the membrane was ruptured by detergent Triton X-100, fast diffusion of EGFP and dextran was recorded with high *k*_efflux_ values of 16.8 ± 2.5 × 10^–3^ s^–1^ and 8.6 ± 1.5 × 10^–3^ s^–1^, respectively (Supplementary Fig. 15). The rapid diffusion also indicates lack of unspecific interaction with the cavity walls.

**Fig. 6:**
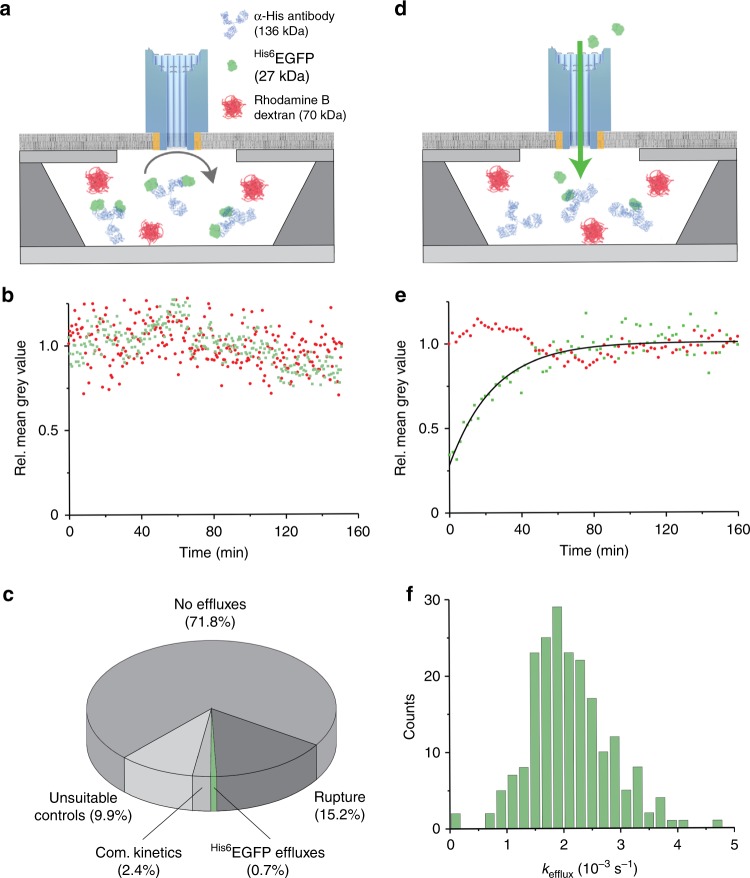
The flux of proteins through single DNA nanopores is modulated by antibodies. **a** Schematic illustration of the antibody recognition assay. Hexahistidine-tagged enhanced green fluorescent protein (^His6^EGFP) (1.0 µM, 27 kDa), Rhodamine B dextran (8.0 µM, 70 kDa), and α-His antibody (0.6 µM, 137 kDa) are sealed inside the chip cavities by supported lipid bilayer formation. After fluxing through the NP pore,  ^His6^EGFP is recognised by α-His antibodies and impeded to move back through the DNA nanopore. **b** Exemplary traces of ^His6^EGFP (green square) and Rhodamine B dextran (70 kDa) (red circle) indicating that efflux of the fluorescent protein is impaired by the EGFP–antibody complex. **c** The results of the recognition assay with 1.1 nM NP are classified into pore-mediated EGFP effluxes, membrane ruptures, complex kinetics, and unsuitable controls without signal changes. **d** Schematic illustration of the antibody-sink experiment. Rhodamine B dextran (8.0 µM, 70 kDa) and α-His antibody (0.6 µM, 137 kDa) are encapsulated inside the chip cavities via supported lipid bilayer formation. After adding to the buffer reservoir ^His6^EGFP (80 nM) and DNA nanopore (1.1 nM), free ^His6^EGFP can diffuse via a DNA nanopore into the cavities where it is recognised and bound by α-His antibody. This leads to an accumulating green fluorescence signal until all antibodies are saturated with fluorescent proteins. **e** Normalised mean grey values of ^His6^EGFP (green square) and Rhodamine B dextran (70 kDa) (red circle) showing accumulation over time for a single cavity, mediated by the antibody sink reaction. **f** Statistical histogram analysis of 208 *k*_efflux_ constants for the antibody-sink experiment. Source data are provided as a Source Data file

Pore-mediated protein translocation was confirmed with a biomolecular recognition assay. Recogniton was achieved by using an IgG antibody with two paratopes to His_6_-tagged EGFP. The antibody (137 kDa) was added to the chip cavities in slight excess over ^His6^EGFP to form a large biomolecular complex and impede pore-mediated efflux of the fluorescent protein (Fig. 6a). Indeed, analysis of hundreds of cavities revealed that 71.8% featured unchanged green fluorescence (Fig. 6b, c) even though a high pore concentration of 1.1 nM was used. Antibodies effectively inhibited pore translocation as only 0.7% of cavities displayed ^His6^EGFP effluxes (Fig. 6c), in comparison to the EGFP-mediated effluxes of 40.4% without α-His antibodies (Fig. 5c). Complex kinetics of ^His6^EGFP efflux were at 2.4% and unsuitable controls of Rhodamine B dextran at 9.9% (Fig. 6c). Membrane ruptures accounted for 15.2% of cavities, which is likely caused by the antibody conservative thimerosal, known to destabilise lipid bialyers^63^. Deliberate rupturing of the membrane with detergent drastically increased the efflux of ^His6^EGFP and Rhodamine B dextran.

To demonstrate that antibody binding can also promote pore-mediated protein translocation, another chip assay was performed. In the sink assay, α-His antibodies are sealed inside the cavities (*trans* side) while ^His6^EGFP is at the *cis* reservoir (Fig. 6d). The flux of ^His6^EGFP through the pore and subsequent binding by α-His antibodies traps the fluorescent protein inside the cavities (Fig. 6d). The reduction  of free ^His6^EGFP thermodynamically drives its influx until all antibody paratopes are saturated. Accumulating proteins against their concentration gradient has not yet been studied with SOI chips. The assay was conducted with 0.6 µM α-His antibody and 8.0 µM control Rhodamine B dextran sealed inside the cavities and 80 nM ^His6^EGFP in the buffer reservoir, followed  by adding 1.1 nM of the DNA pore to induce flux. In support of a successful sink reaction, a strong fluorescence increase for ^His6^EGFP was seen  in cavities after 4 h (Supplementary Fig. 16). The ^His6^EGFP signal rose up to fivefold (Fig. 6e, green symbols), whereas the signal for control Rhodamine B dextran stayed constant (Fig. 6e, red symbols). In total, 208 influx traces were analysed showing a rise in ^His6^EGFP fluorescence (Fig. 6f). As expected, membrane rupturing with a detergent drastically increased the efflux of ^His6^EGFP and Rhodamine B dextran. Transport was also established with another molecular cargo, a fluorophore-labelled polymer, using a bulk-transport assay with giant unilamellar vesicles (GUVs) (Supplementary Fig. 18).

## Discussion

This report has pioneered the translocation of folded proteins through a synthetic membrane-spanning DNA nanopore which is of relevance for biophysics, biosensing, and DNA nanotechnology. Transport through the 50 nm^2^ wide pore lumen was analysed in high-throughput fashion with massively parallel single-channel readout, in addition to electrical recordings. The dual analysis revealed the influence of nanoconfinement on transport and the 20-fold slower protein speed for diffusion- compared to electric field-driven transport. The study thereby provides a step-change to existing biogenic and synthetic channels to advance understanding of the biophysics of transport as well as to promote nanopore-based biosensing. Previously, transport of folded proteins has not been achieved with membrane- but with inorganic solid-state pores^7,19,64^. However, the latter are not compatible with biological bilayers or synthetic membranes that are commonly used in biosensing platforms. Membrane-spanning and wide DNA pores address this shortcoming by using a highly modular design principle that can adjust pore diameter, which is a quality difference compared to protein pores. Larger DNA pores may be assembled from multiple smaller DNA origami units. Molecular receptors capable of specifc analyte binding may also be installed in the pore lumen, based on predictable DNA hybridisation^65^.

A DNA nanopore compatible with biosensing also advances DNA nanotechnology which strives to create nanodevices with functions relevant for applications outside the field^34^. The DNA membrane pore furthermore supports biomimetic DNA nanotechnology which replicates the function of natural proteins with simpler-to-engineer DNA nanostructures such as cytoskeletal-like membrane-shaping scaffolds^66–68^. To further mimic nature, the  nanopore could be turned into a molecular gate to regulate the flow of proteins across membranes^39,69^ for drug delivery nanodevices^70^ composed of stable vesicles with biocompatible polymer walls^71,72^. Another exciting option is to build a molecular machine that selectively moves cargo across membranes^73^, something which is supported by the antibody-sink reactions. These biomimetic structures are of interest in basic research, biotechnology and biomedicine^74,75^. In conclusion, our study overcomes limitations of biogenic and synthetic membrane nanopores and opens up several applications in science and technology.

## Methods

### Materials

Native and cholesterol-labelled DNA oligonucleotides with a tri(ethylene glycol)(TEG) linker were purchased from Integrated DNA Technologies (Leuven, Belgium) or ATDbio (Southampton, United Kingdom) on a 1 μmol scale with desalting or HPLC purification, respectively. 1,2-dioleoyl-*sn*-glycero-3-phosphoethanolamine (DOPE), 1,2-dioleoyl-*sn*-glycero-3-phosphocholine (DOPC), and 1,2-diphytanoyl-sn-glycero-3-phosphocholine (DPhPC) were procured from Avanti Polar Lipids (Alabaster, AL). m13mp18 DNA was obtained from New England Biolabs (Ipswitch, United Kingdom). PEG^350^-FAM was procured from Chem Quest (United Kingdom). All other reagents and solvents were purchased from Sigma-Aldrich unless stated otherwise.

### Nanopore design

The DNA origami nanopore NP was designed using the square lattice version of the CaDNAno software^43^. To assess rigidity in the structural design, several cycles of strand routing with caDNAno and CanDo^76^ modelling were conducted. The 7249 nt-long single-stranded m13mp18 DNA was selected as scaffold strand. The rendering of the DNA nanopore and the 2D DNA map are in Supplementary Figs. 1 and 2, respectively. In the design, lipid anchors are attached to the pore via DNA oligonucleotides that carry cholesterol at the 5′ or 3′ terminus. These cholesterol-modified anchor strands hybridise via adaptor oligonucleotides to the pore. The adapter-mediated binding enables limitation of the number of expensive cholesterol-modified oligonucleotides to two. The DNA sequences of staple strands, adaptor strands, and cholesterol-modified anchor strands are provided in Supplementary Table 1.

### Assembly

For the assembly of the DNA nanopores,  NP^ΔC^ was  annealed in a one-pot reaction containing 1× TAE buffer, supplemented with 14 mM MgCl_2_, and a mixture of m13mp18 scaffold and staples at final concentrations of 4.2 and 100 nM, respectively. Assembly was conducted using a 8-day-long protocol involving a first annealing phase from 80 °C to 60 °C at a cooling rate of 1 °C per 5 min, and a second phase from 60 °C to 20  °C at a rate of 1 °C per 300 min. To form NP with cholesterol lipid anchors, NP^ΔC^, purified by size-exclusion chromatography (SEC) (see below), was mixed with cholesterol-modified anchor oligonucleotides (1.1 eq. strand per binding site at the pore, up to 24 sites) and incubated at 30 °C for 12 h.

### Agarose gel electrophoresis of DNA nanopores

The assembly products NP and NP^ΔC^ were analysed using 1.5% agarose gel electrophoresis in standard 1× TAE buffer, optionally supplemented with 0.015% SDS. DNA pore samples (10 μL) were mixed with 6× gel loading buffer (2 μL) and then loaded into the wells. Gels were run at 70 V for 1 h at 8 °C. A 1000-base-pair marker (New England Biolabs) was used as the reference standard. DNA bands were visualised by staining with ethidium bromide solution and ultraviolet illumination. SDS containing gels were washed with deionised water for 20 min prior to staining. To analyse the interaction of NP with membranes, SUVs were formed. Chloroform solutions of DOPE (0.3 mmol, 22.3 µL) and DOPC (0.7 mmol, 110 µL) were mixed, and added to an oven-dried round bottom flask (10 mL), followed by removal of the solvent under vacuum using a rotary evaporator for 20 min. To form vesicles, a solution of 0.3 M KCl, 15 mM Tris, pH 8.0 (1 mL) was added, and the suspension was sonicated for 20 min at RT. SUV preparations were stored at 4 °C and used within one week. Before experimentation, the SUV solution was vortexed for 2 s. For agarose gel electrophoretic analysis of NP nanopores with SUVs, the same gel conditions as described above were used, except that SDS was omitted and gels were run at 40 V. Pores (15 µL, 1 µM, 0.3 M KCl, 15 mM Tris, pH 8.0) were incubated with SUVs (15 µL, 1 mM, 0.3 M KCl, 15 mM Tris, pH 8.0) for 30 min at 37 °C. Blue loading dye (6×, no SDS, 10 µL) was added to the mixture and loaded onto the gel (30 µL).

### Purification

Assembled NP^ΔC^ nanopores were purified from excess staples using SEC using an ÄKTA purifier 100/10 fitted with a Superdex 200 10/300 GL column (GE Healthcare), using a flow rate of 0.5 mL per min at 8 °C. Elution was monitored with UV–vis absorption at 260, 280, and 295 nm, and fractions containing the folded DNA pore were pooled.

### Transmission electron microscopy

Samples of membrane-inserted DNA nanopores were prepared by incubating NP at a final concentration of ~1 nM with the pre-formed SUVs (total lipid concentration ~10 µM, DOPC/DOPE = 7: 3 mol%) in 1× TAE buffer supplemented with 0.3 M NaCl for 30 min at RT. The purified NP^ΔC^ pore or the mixture of NP and SUVs (6 µL of sample solution) were added onto glow discharge-treated TEM grids and stained with 0.5% uranyl acetate solution. TEM analysis was performed on a JEM-2100 electron microscope (JEOL) operated at 200 kV and images were acquired with an Orius SC200 camera.

### Nanopore current recordings

For planar lipid bilayer electrophysiological current measurements, integrated chip-based, parallel bilayer recording setups (Orbit 16 and Orbit Mini; Nanion Technologies, Munich, Germany) with multielectrode-cavity-array (MECA) chips (IONERA, Freiburg, Germany) were used^10,39,47^. Bilayers were formed by painting DPhPC dissolved in octane (10 mg mL^–1^). The electrolyte solution was 1 M KCl and 10 mM HEPES, pH 8.0. For pore insertion, a 2:1 mixture of cholesterol-anchored DNA nanopore NP and 0.5% OPOE (n-octyloligooxyethylene, in 1 M KCl, 10 mM HEPES, pH 8.0) was added to the *cis* side of the bilayer. Successful incorporation was observed by detecting current steps. The Orbit Mini was in this case used for all protein translocation studies and the Orbit 16 for all other current measurements. The Orbit 16 current traces were Bessel-filtered at 2.873 kHz and acquired at 10 kHz with an EPC-10 patch-clamp amplifier (HEKA Elektronik, Lambrecht/Pfalz, Germany) applying the PATCHMASTER software (HEKA Elektronik). The Orbit Mini current traces were not Bessel-filtered and acquired at 10 kHz, using Element Data Recorder software (Element s.r.l., Italy). Single-channel analysis was performed using Clampfit (Molecular Devices, Sunnyvale, CA, USA). The theoretical conductance of NP was calculated using the following equation, in accordance with^37^:

1 $$G = \kappa \frac{{{\mathrm{\pi }}d^2}}{{4L + {\mathrm{\pi }}d}},$$

where *κ* is the electrical conductivity (equal to 10.86 Sm^–1^ for 1 M KCl at 25 °C), *d* is the diameter, and *L* the length of the pore.

### Release assays with fluorophore-filled vesicles

Giant unilamellar vesicles (GUVs) were formed by adding a solution of DOPE (0.3 μmol, 50 μL) and DOPC (0.7 μmol, 550 μL) to an oven-dried round bottom flask (10 mL), and the solvent removed under vacuum using a rotary evaporator for 20 min, followed by ultra-high vacuum for 3 h. A solution of sorbitol (1 M, 1 mL) containing PEG350-FAM (10 μM) was added to the flask, and the solution was sonicated for 30 s to form fluorophore-filled vesicles. After 20 min, a portion of the GUV suspension (1 μL) was added to PBS (200 μL) within an eight-well glass chamber (LabTek). After allowing the vesicles to settle for 5 min, a mixture of NP/OPOE (100 μL SEC-purified NP/12.5 μL 0.5% OPOE with 37.5 μL PBS) or cholesterol strands/OPOE (100 μL anchor-cholesterol strand/12.5 μL 0.5% OPOE, 37.5 μL PBS) was added. The fluorescence images were collected after 30 min using a confocal laser scanning microscope (FV-1000 Olympus), ×60 oil objective, excitation at 515 nm, and the appropriate emission filters using identical settings for all vesicles.

### Chip fabrication

The SOI chips (13 × 13 mm^2^) comprised a 3.0 ± 0.5 µm silicon (100) device layer, 100 ± 10 nm buried oxide (BOX), and a 380 ± 15 µm undoped silicon (100) handling substrate. These chips were coated by stoichiometric Si_3_N_4_ (thickness ∼50 nm) on both sides, using low-pressure chemical vapour deposition. A square window (1.8 × 1.8 mm^2^) was then opened in the Si_3_N_4_ layer at the centre of the chip’s backside via optical lithography (Shipley S1818 photoresist) and reactive ion etching (RIE) using a C_4_F_8_/O_2_ gas mixture, 150 W power, and a duration of 85 s. Subsequently, anisotropic wet etching was performed in aqueous potassium hydroxide solution (20 wt% KOH, 80 °C, 3 h) to remove the bulk Si layer at the backside, thereby forming a large pyramidal pit truncated at the BOX layer. The next fabrication step yielded arrays of cavities opened by a nanoscale orifice of 80 nm diameter. The square arrays (120 × 120 µm^2^, with a pitch of 10 µm) were patterned by electron beam lithography (EBL, e_LiNE system, Raith, Dortmund, Germany) using EBL resist AR-P 6200 (Allresist, Strausberg, Germany), an acceleration voltage of 30 kV, a beam current of ∼30_;_pA, and developer AR 600-546 (Allresist, Strausberg, Germany). Subsequently, the nano-orifice array resist pattern was transferred into the Si_3_N_4_ layer by another RIE step (C_4_F_8_/O_2_, 150 W, 85 s). The final, homogenous array of 14,400 nano-orifice cavities with volumes ∼50 fL was obtained by a second anisotropic wet etching, now of the Si device layer, in KOH solution (15 wt%, 50 °C, 3.5 h). Each individual cavity features the shape of an inverted pyramid, whose tip is truncated at the BOX layer (i.e. the transparent cavity bottom).

### Liposome preparation for chip translocation assay

Liposomes used to obtain supported lipid bilayers (SLB) were prepared as follows. Briefly, a dried lipid film of DOPC and DOPE at a molar ratio of 7:3 was resuspended in 1× TAE buffer supplemented with 14 mM MgCl_2_ pH 8.3, followed by sonication for 20 min. Afterwards, the lipid suspension was extruded 21 times through polycarbonate filters (100 nm) using a LiposoFast-Basic extruder (AVESTIN, Mannheim, Germany), followed by five freeze–thaw cycles.

### SOI chip preparation

The silicon chips were cleaned in oxygen plasma for 2 min at 0.3 mbar and 80% power with the Diener Electronics plasma cleaner (Ebhausen, Germany) followed by gluing the activated surface onto eight-well adhesive slides (ibidi, Planegg/Martinsried, Germany). Afterwards, the chip was washed with ethanol followed by 1× TAE buffer supplemented with 14 mM MgCl_2_ pH 8.3. Depending on the experiment, solutions of ^His6^EGFP, Rhodamine B dextran (70 kDa), or α-His antibodies were used followed by adding the liposome suspension (1 mg mL^–1^, incubation 1 h). Afterwards, the buffer reservoir was washed several times with 1× TAE/14 mM MgCl_2_ before the single-transport recordings were carried out. Sealing efficiencies were determined by dividing the EGFP/dye filled cavities by the total cavities per field of view. ^His6^EGFP was purified as described^77^ Rhodamine B dextran (70 kDa) was purchased from Thermo Fisher Scientific (Darmstadt, Germany), and α-His antibody was obtained from Abcam (Berlin, Germany).

### Single-transport optical recordings

Optical single-pore recordings were performed using the SOI chips and readout with a confocal laser scanning microscope (CLSM) LSM 880 (AxioObserver from Zeiss, Jena, Germany) equipped with a Plan-Apochromate ×20/0.8 M27 air objective. Different concentrations of the NP DNA nanopore were applied and time-lapse images were recorded with varying time intervals dependent on the experiment. Each assay was ceased by solubilizing the SLB with the addition of Triton X-100 4% (v/v) at a final concentration of 0.4%. The termination of the assay was recorded in time-lapse images via CLSM.

### Analysis of single-transport optical recordings

The optical time-lapse images were processed with the Zen 2.1 black software by Zeiss, followed by drift corrections, region of interest analysis, and mean grey value extraction by ImageJ. The monoexponetial translocation kinetics were fitted with the Nanocal software (Nanospot GmbH), and the resulting fist-order rate constants (*k*_efflux_) were statistically analysed with Origin 9.1 Pro (OriginLab), including Gaussian fitting. The antibody-sink reaction was analysed in a similar manner; however, the onset of the EGFP increase traces was fitted by a linear function due to the constant flux gradient mediated by the antibodies and due to the similarity in the efflux rate.

## Supplementary information

Supplementary Information

Description of Additional Supplementary Files

Supplementary Data 1

Source Data

## Data Availability

The source data underlying Fig. 2, Fig. 3b, Fig. 3c, Fig. 4c Fig. 5b–e, Figs. 6b, 6c, 6e, 6f, and Supplementary Figs. 7b, 8b, 10a-f, 11c-f, 14, 15 and 17 are provided as a Source Data file. The data underlying the results of this study are available from the corresponding authors upon reasonable request.
