# Supplementary material for: Synthetic protein-conductive membrane nanopores built with DNA
Source: Nat Commun. 2019 Nov 4;10:5018. doi: 10.1038/s41467-019-12639-y (PMC6828756; doi:10.1038/s41467-019-12639-y)
Supplement: Supplementary file 1 — Supplementary Information [file 41467_2019_12639_MOESM1_ESM.pdf]

Supplementary Information

Synthetic protein-conductive membrane nanopores built with DNA

Diederichs et al.

Supplementary Figures

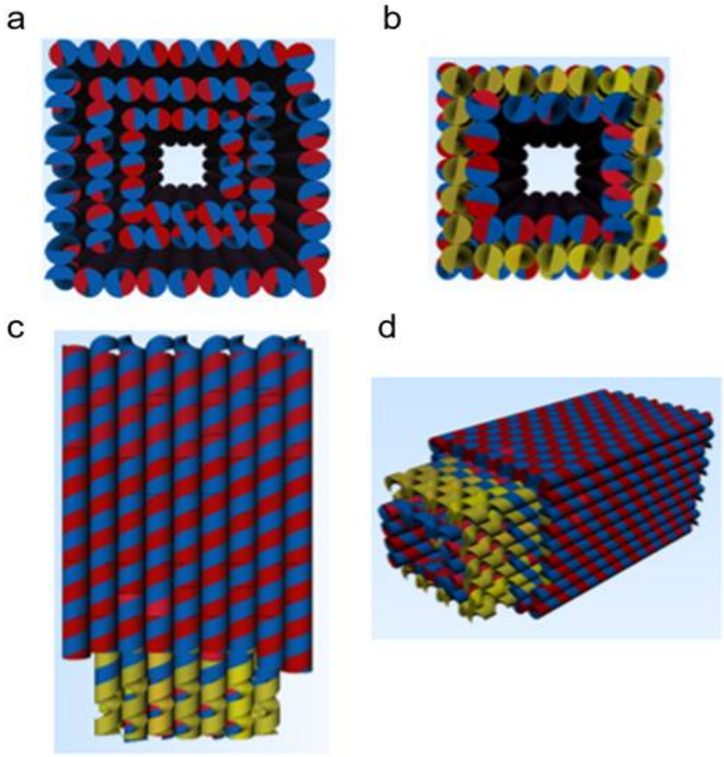

Supplementary Figure 1. Rendering images of DNA nanopores. Nanopore (NP) was designed using CaDNAno software and is shown from the top (**a**), bottom (**b**), side (**c**), and angular side view (**d**). The cylinders represent DNA duplexes which are composed of scaffold strand (blue) and the staple oligonucleotides (red). Yellow strands are adaptor oligonucleotides which hybridize with the unpaired single-stranded portion to cholesterol-modified anchor oligonucleotides (not shown).

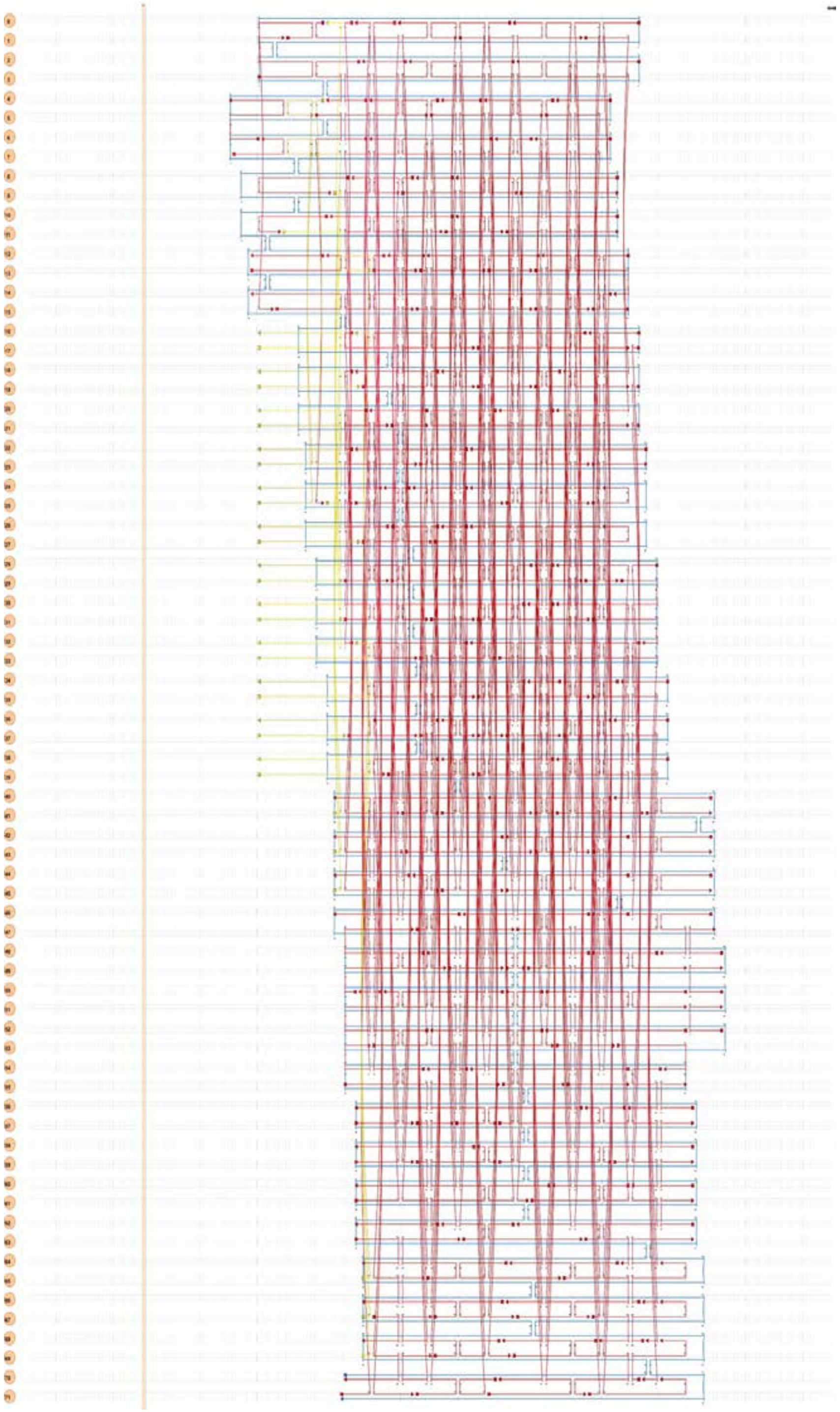

Supplementary Figure 2. 2D DNA map of the nanopore $NP^{\Delta C}$. The scaffold strand is shown in blue and staple strands are in red. Yellow indicates adaptor strands which hybridize with a portion of their sequence to the cholesterol-modified anchor oligonucleotides. 5' and 3' termini of DNA strands are represented as a squares and triangles, respectively. The duplexes are numbered at the left.

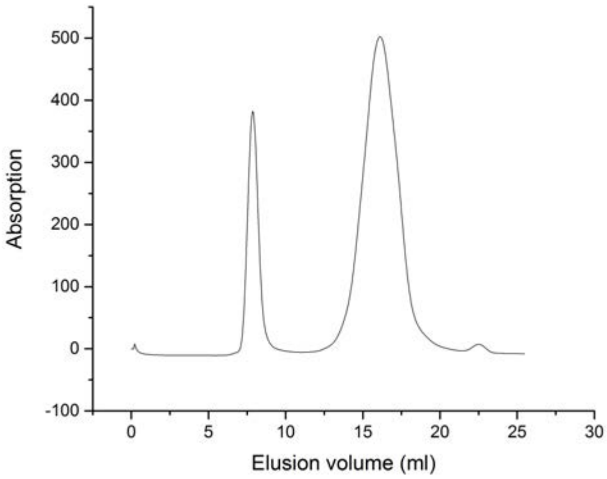

Supplementary Figure 3. Purification of DNA nanopores. Size exclusion chromatography trace of an assembly mixture containing the folded DNA nanopore (elution volume of 7.88 mL) and excess staple strands (elution volume of 16.13 mL). The DNA pore was detected by determining absorption at 260 nm.

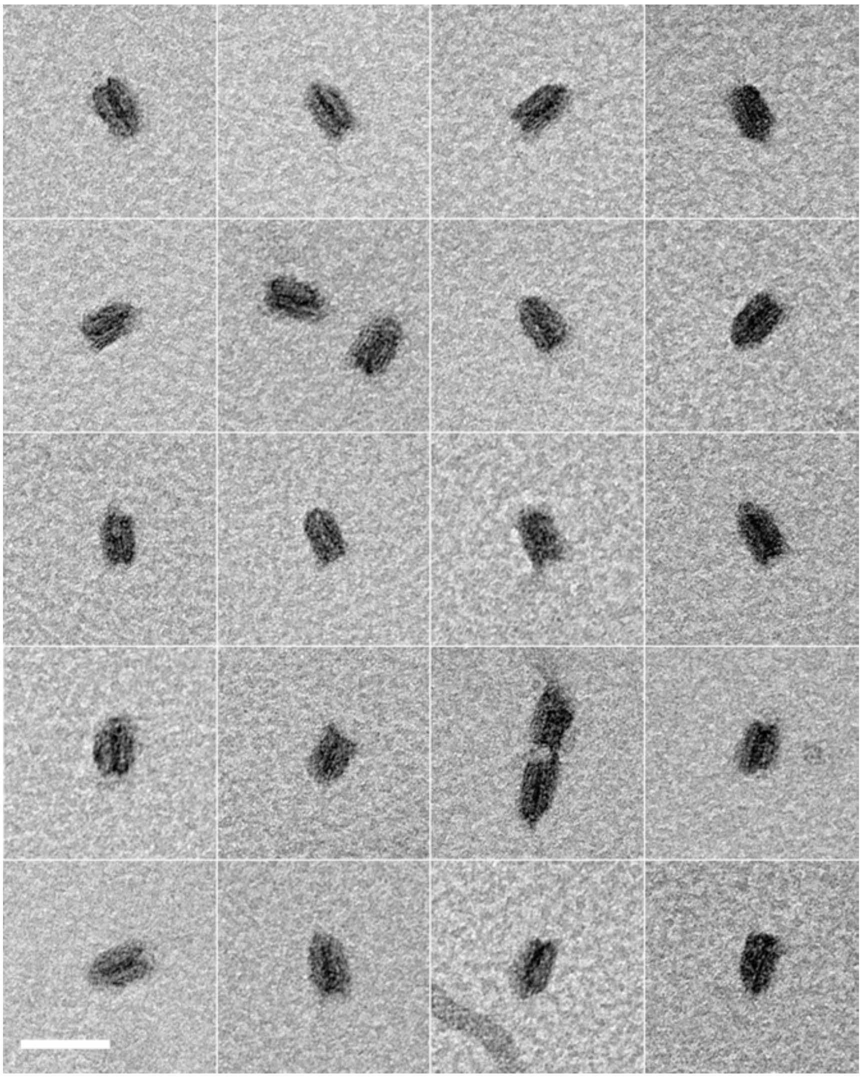

Supplementary Figure 4. Confirming the structure of DNA nanopores. Representative transmission electron microscopy (TEM) images of negatively stained $NP^{\Delta C}$ pores. Scale bar, 50 nm.

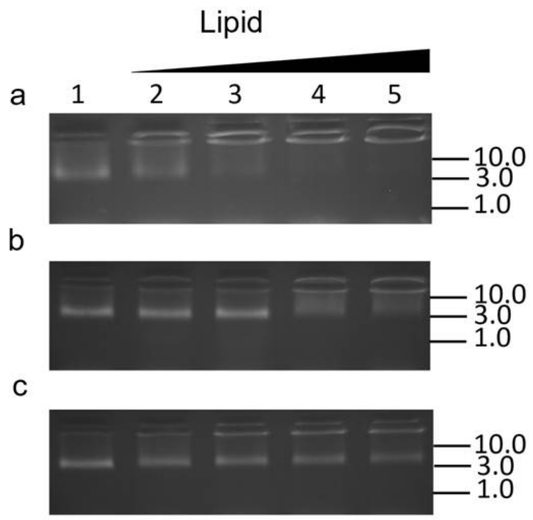

Supplementary Figure 5. Gel electrophoretic analysis of the interaction between cholesterol-tagged DNA nanopores and lipid vesicles. **a** The DNA nanopores carried the lipid anchor at all 24 possible membrane-spanning duplexes. This corresponds to 12 DNA oligonucleotides with the cholesterol TEG modification attached at the 5' terminus and 12 at the 3' terminus. **b, c** Gels for pores with (**b**) 12 lipid anchors at 5' termini, and (**c**) 12 lipid anchors at 3' termini. The lipid concentrations are 0.42 mM (lane 2), 0.58 mM (lane 3), 0.67 mM (lane 4), 0.75 mM (lane 5). The position and bp length of the dsDNA markers are given at the right of the gels. Source Data are provided as a Source Data file.

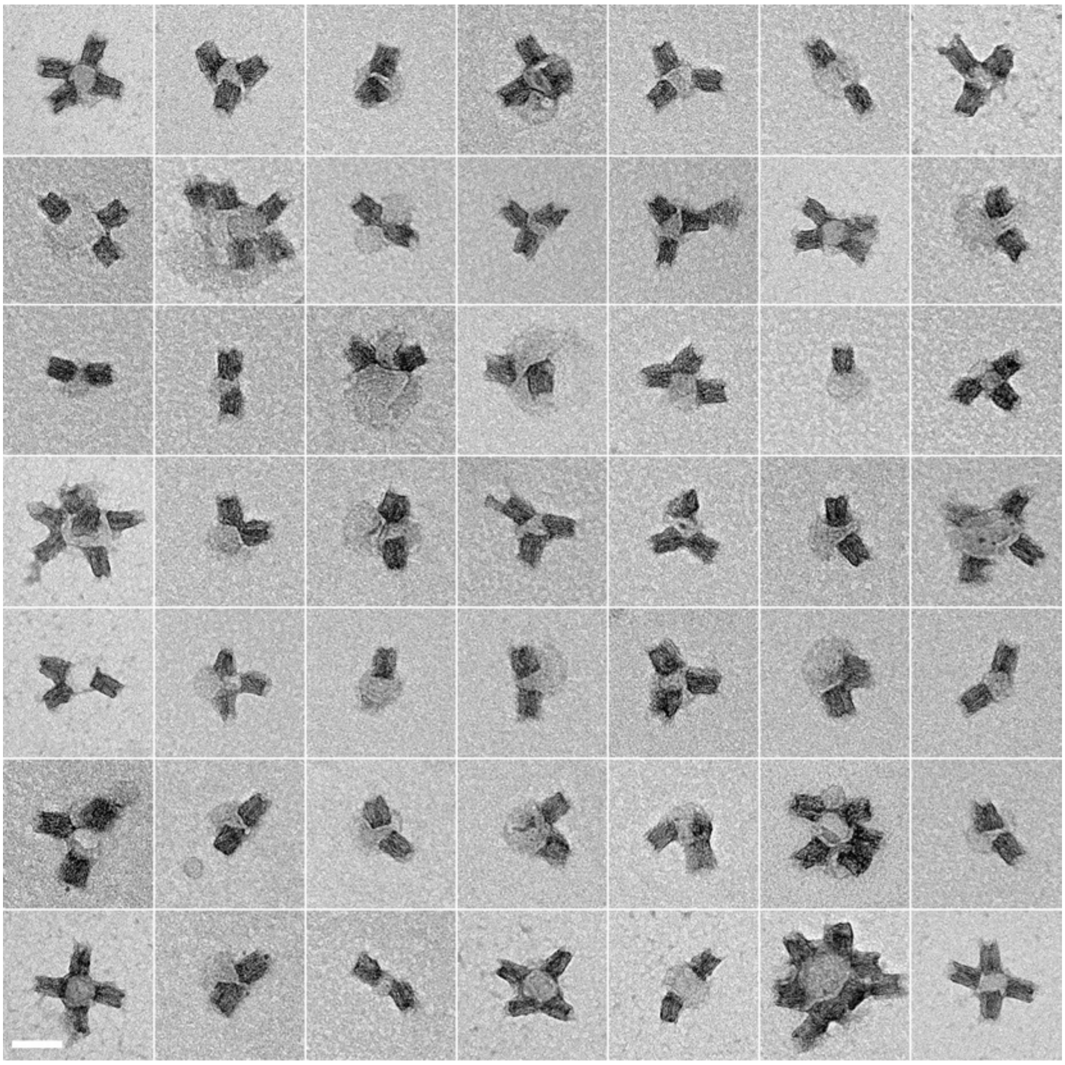

Supplementary Figure 6. NP pores insert into membrane vesicles. Representative TEM images of negatively stained NP pores inserted into small unilamellar vesicles. Scale bar, 50 nm.

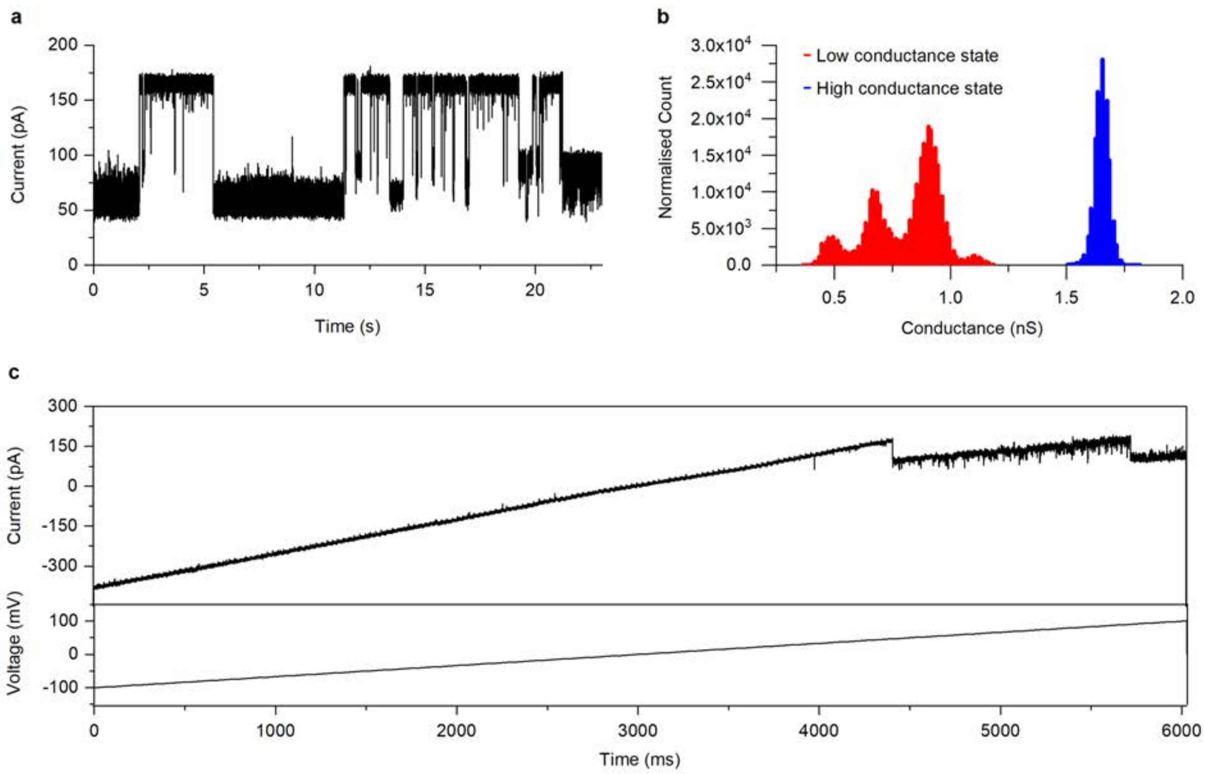

Supplementary Figure 7. Subconductance states of NP at high voltages. **a** Representative ionic current trace of a single NP at +100 mV relative to the *trans* side of the membrane. The trace displays subconductance states at higher voltages. Electrophysiological buffer composed of 1 M KCl, 10 mM HEPES, pH 8.0. **b** All-point current frequency histogram of one NP, obtained at +100 mV showing low conductance (blue) and high conductance (red) states. **c** Representative ionic current trace in which current has been ramped from -100 mV to +100 mV over 6 s. Electrical recordings were measured using the Orbit mini device which is grounded at the *trans* chamber. Source data are provided as a Source Data file.

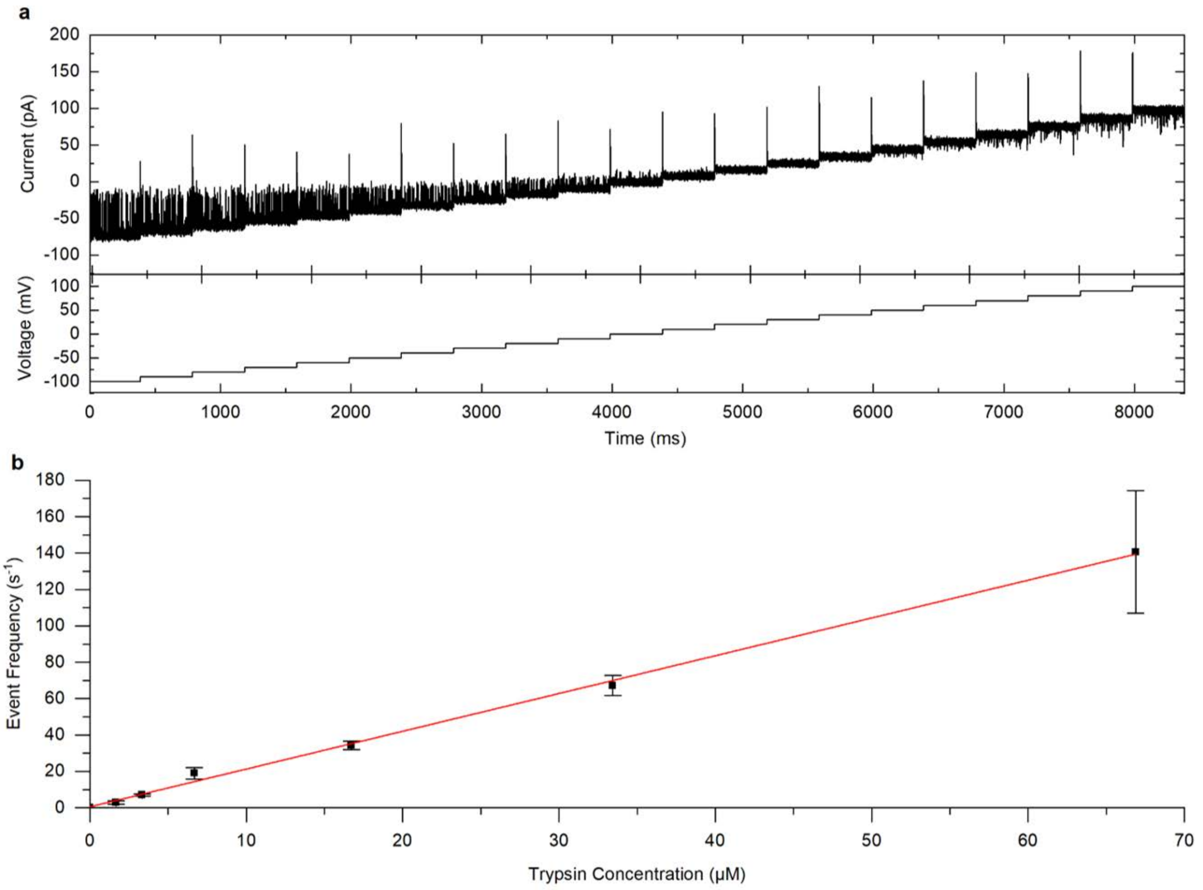

Supplementary Figure 8. Trypsin-induced blockade events of DNA nanopores. **a** Current trace of a single NP nanopore at voltage steps recorded in the presence of 66 μM trypsin at the *cis* side. The electrophysiological buffer was 1 M KCl, 10 mM HEPES, pH 8.0. The voltage was ramped from -100 mV to +100 mV in 10 mV steps. **b** Scatter plot showing the relationship between trypsin concentration and average event frequency ± SEM at -100 mV. Averages were obtained from a minimum of three independent single-channel recordings. Electrical recordings were measured using the Orbit mini device which is grounded at the *trans* chamber. Source data are provided as a Source Data file.

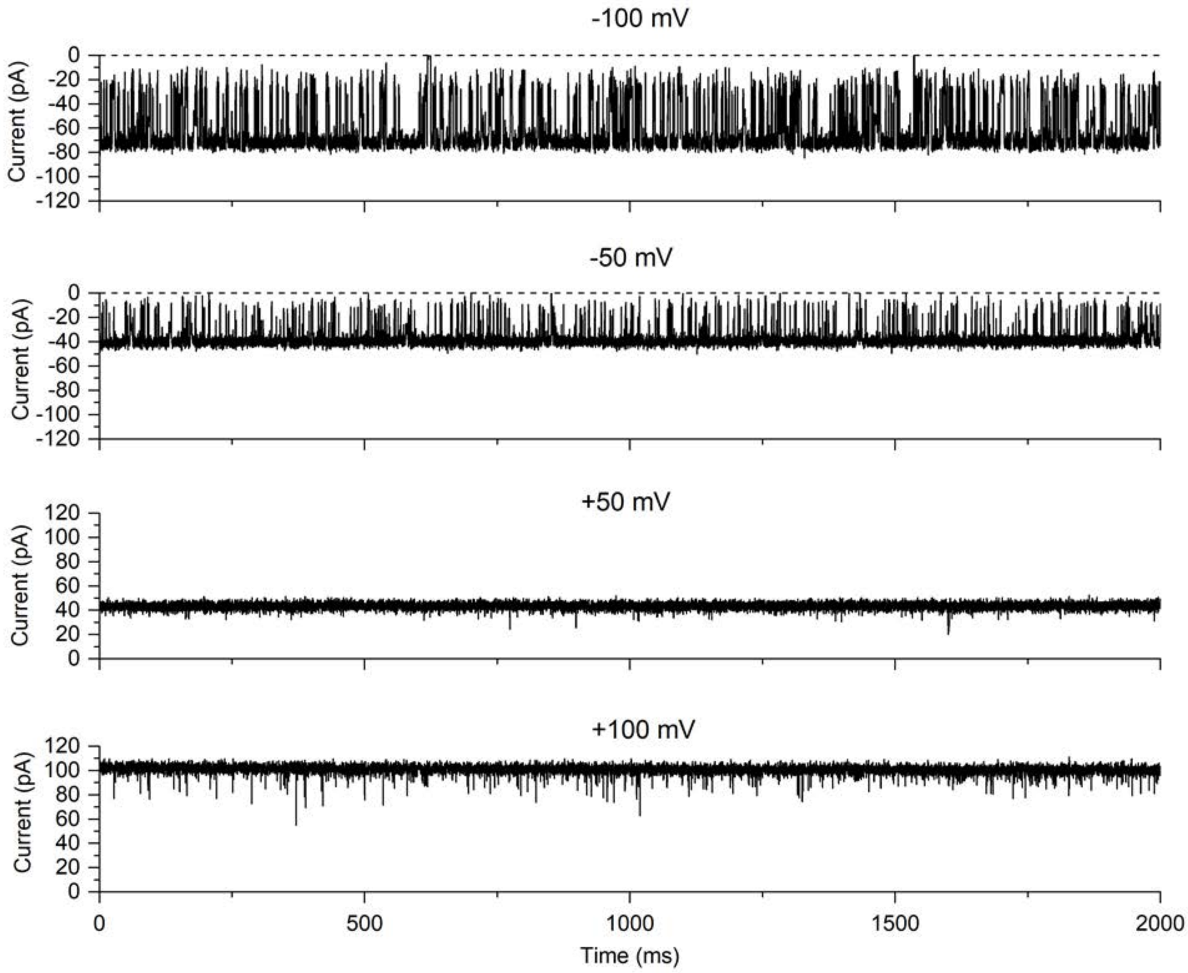

Supplementary Figure 9. Voltage-dependent transport of trypsin through a NP nanopore. Voltages were +50 mV, +100 mV, -50 mV and -100 mV. The electrophysiological buffer was 1 M KCl, 10 mM HEPES, pH 8.0. The single-channel current traces were recorded in the presence of 66 µM trypsin at the *cis* side leading to current blockades. Electrical recordings were obtained using the Orbit mini device which is grounded at the *trans* chamber.

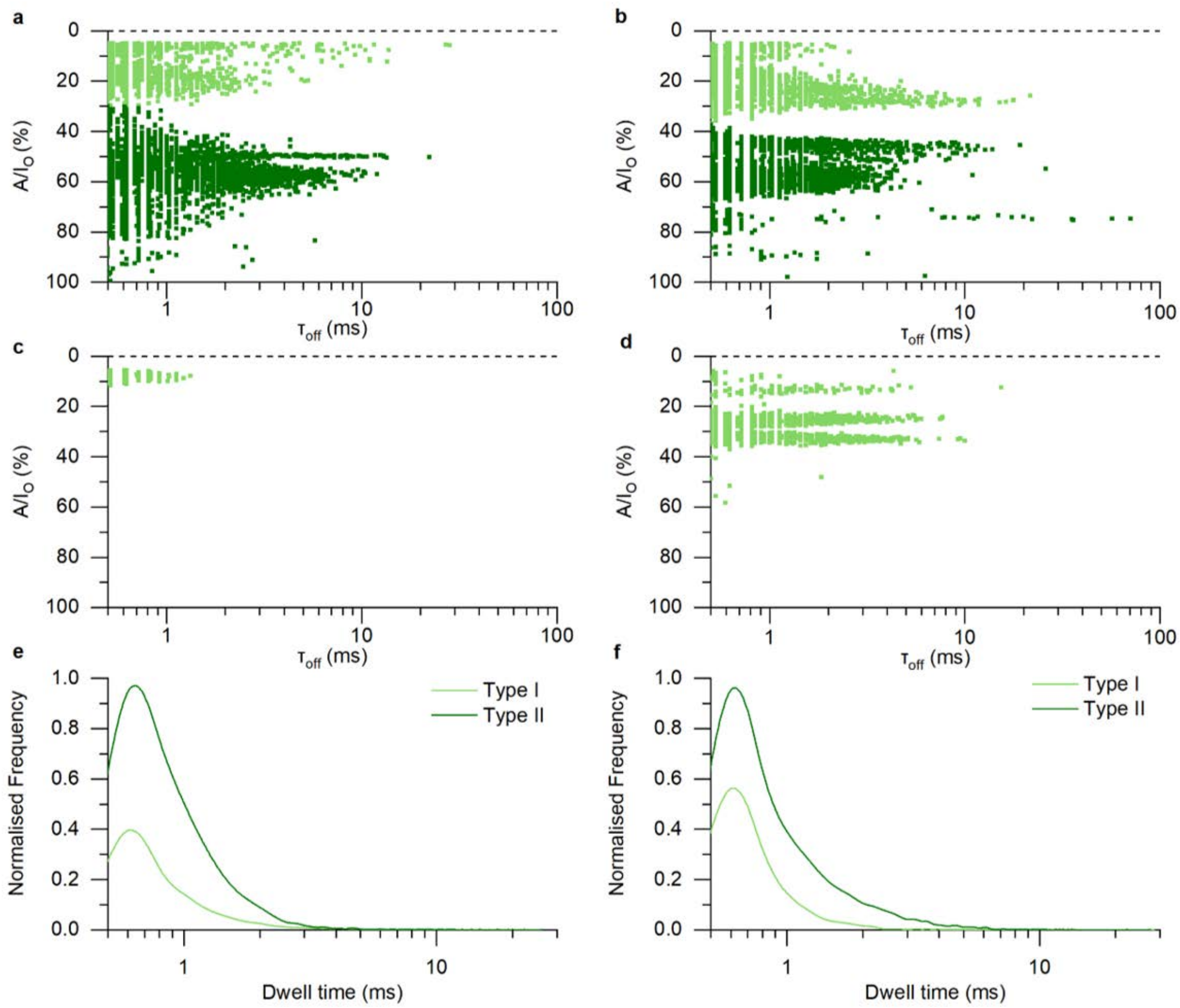

Supplementary Figure 10. The transport of trypsin through NP nanopores is voltage-dependent. Each point in the scatter plot represents an individual encounter event of a protein molecule with a single DNA nanopore. Each event is characterised by its duration, τ_{off}, and amplitude, *A*. The events were obtained using single-channel current recordings with 66 µM trypsin in the *cis* chamber. The electrophysiological buffer was 1 M KCl, 10 mM HEPES, pH 8.0. **a** At -50 mV, the scatter plot comprises 7282 data points, **b** at -100 mV 8839 data points, **c** at +50 mV 656 data points, **d** and at +100 mV 3556 data points. The data in scatter plots represent protein translocation events from three independent recordings. **e** Dwell-time histogram of type I and type II events at -100 mV. **f** Dwell-time histogram of type I and type II events at -50 mV. Electrical recordings were obtained using the Orbit mini device which is grounded at the *trans* chamber. Source data are provided as a Source Data file.

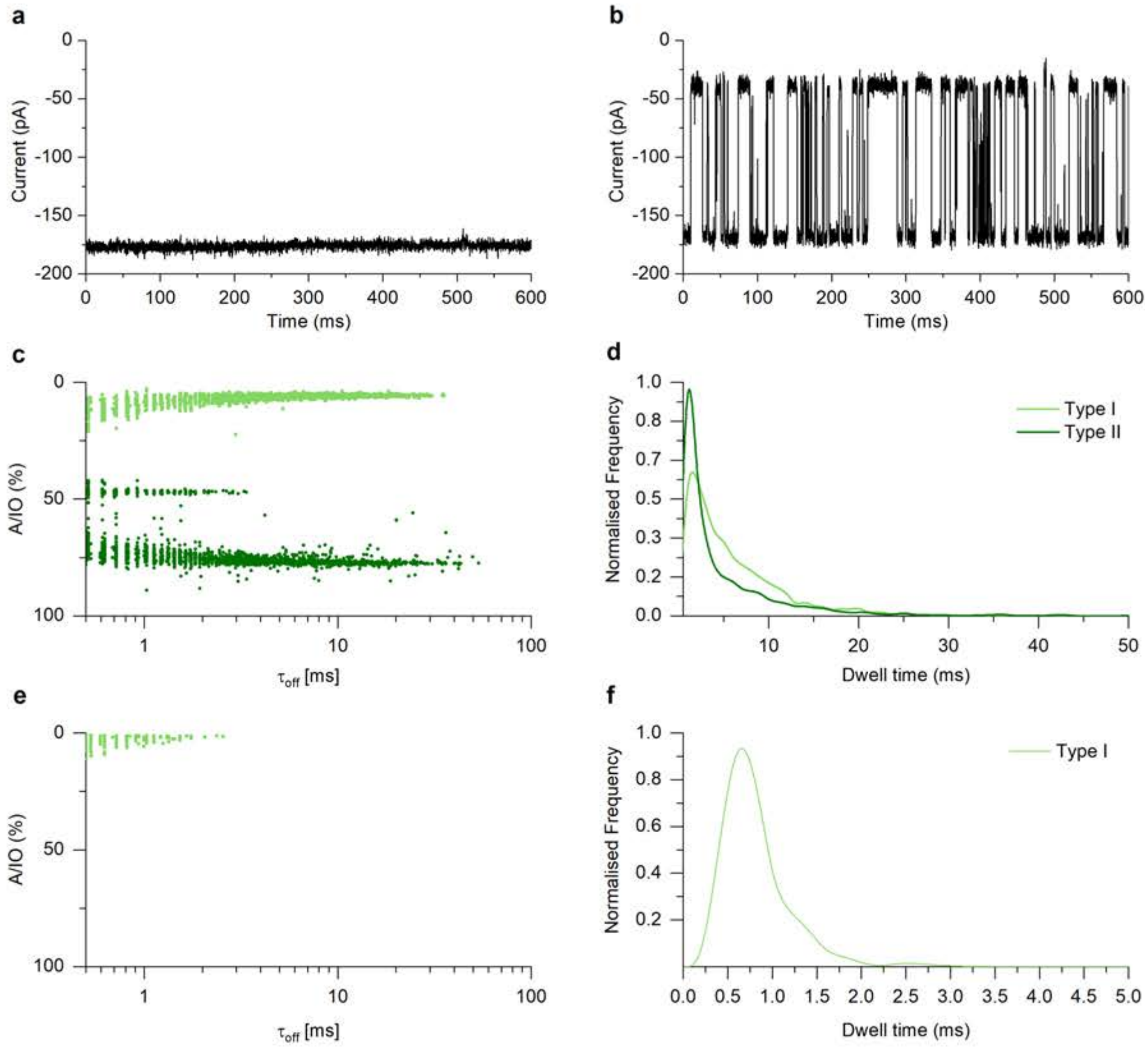

Supplementary Figure 11. The translocation of green fluorescent protein is pH- and voltage-dependent. **a** A representative single-channel current trace recorded at -100 mV in pH 8.0 where green fluorescent protein (GFP) (200 nM) was added to the *cis* side. **b** A single-channel current trace recorded at -100 mV in pH 5.0, in which the same concentration of GFP leads to current blockades. **c** Scatter plot showing event duration, τ_{off}, and amplitude, A, for single-channel current recordings under conditions as in panel b. Each point in the diagram represents an individual encounter event of a protein molecule with a DNA nanopore. The scatter plot comprises 4,500 data points from three independent recordings. **d** Dwell-time histogram of type I and type II events at -100 mV. **e** Scatter plot showing τ_{off} and A for single-channel current recordings with GFP in electrophysiological buffer of pH 5.0 at +100 mV. The scatter plot comprises 274 data points. **f** Dwell-time histogram of type I events at +100 mV. Electrical recordings were obtained using the Orbit mini device which is grounded at the *trans* chamber. Source data are provided as a Source Data file.

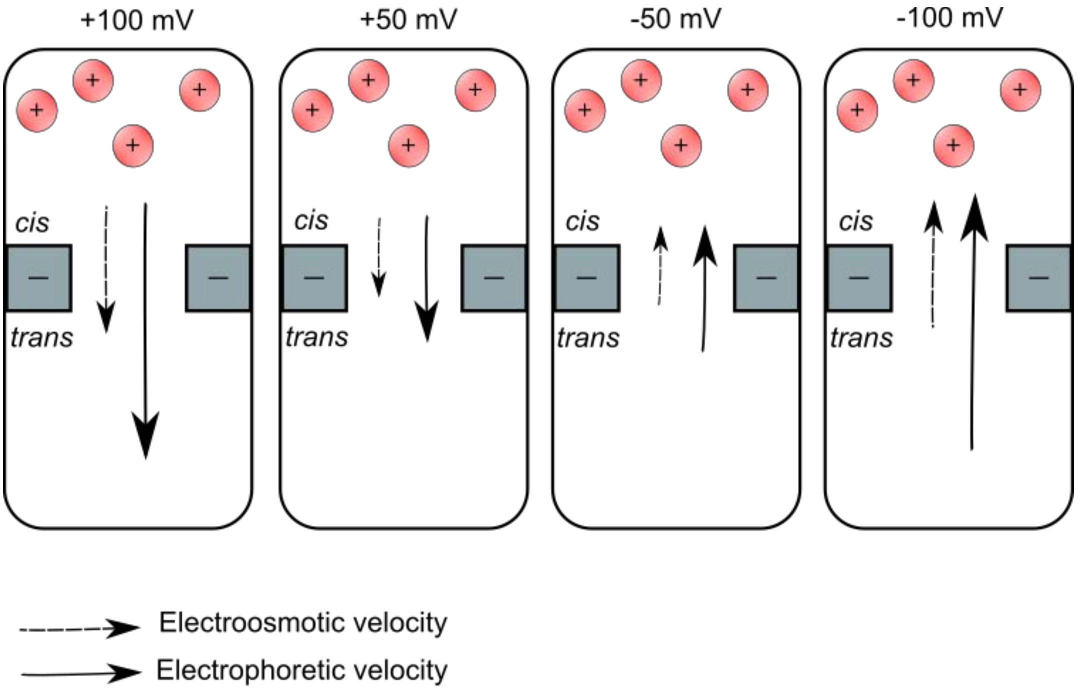

Supplementary Figure 12. Factors influencing protein transport. Schematic illustration of predicted electrophoretic and electroosmotic forces acting on trypsin (red) when moving through the DNA nanopore (grey) at different membrane potentials. The potentials are given for the *trans* side grounded.

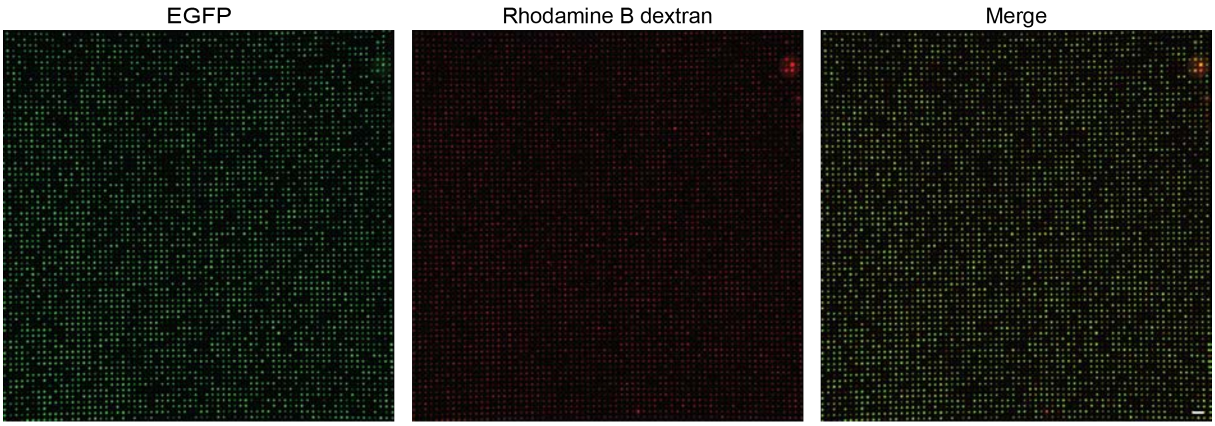

Supplementary Figure 13. Fluorophore sealing in silicon-on-insulator chip cavities. Enhanced green fluorescent protein (EGFP) (5 μM, 27 kDa) and Rhodamine B dextran (8 μM, 70 kDa) were encapsulated inside the silicon-on-insulator microcavities *via* liposome spreading, followed by buffer exchange to remove lipidic and fluorophore excess indicating the high-throughput of the optical translocation recordings. Scale bar, 20 μm.

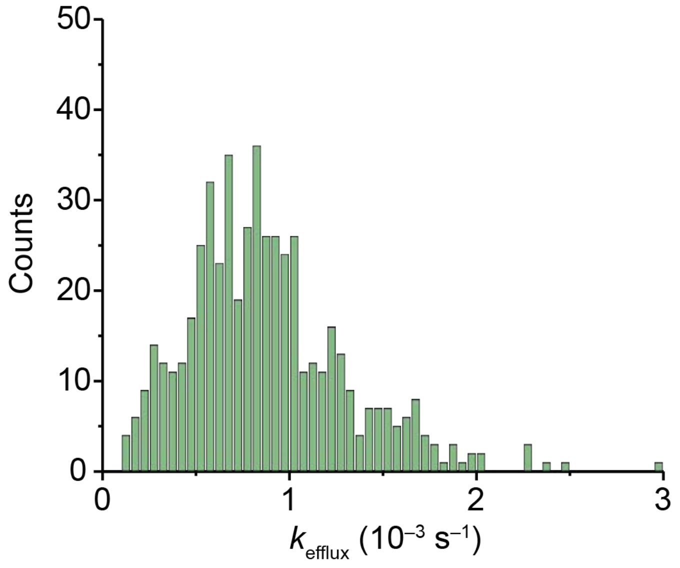

Supplementary Figure 14. Translocation kinetics through DNA nanopores added at a concentration of 3.2 nM. Enhanced green fluorescent protein (EGFP) (5 µM, 27 kDa) and Rhodamine B dextran (8 µM, 70 kDa) were sealed inside the microcavities *via* liposome spreading. After removal of the lipidic and fluorescent excess by buffer exchange, DNA nanopores were added to a final concentration of 3.2 nM. The flux measurements are summarised in a histogram with 523 flux traces that featured a monoexponential decay for the EGFP channel and constant signals in the Rhodamine B dextran (70 kDa) channel. The heterogeneous distribution in the histogram indicates undesirable multiple nanopore insertions per cavity. Source data are provided as a Source Data file.

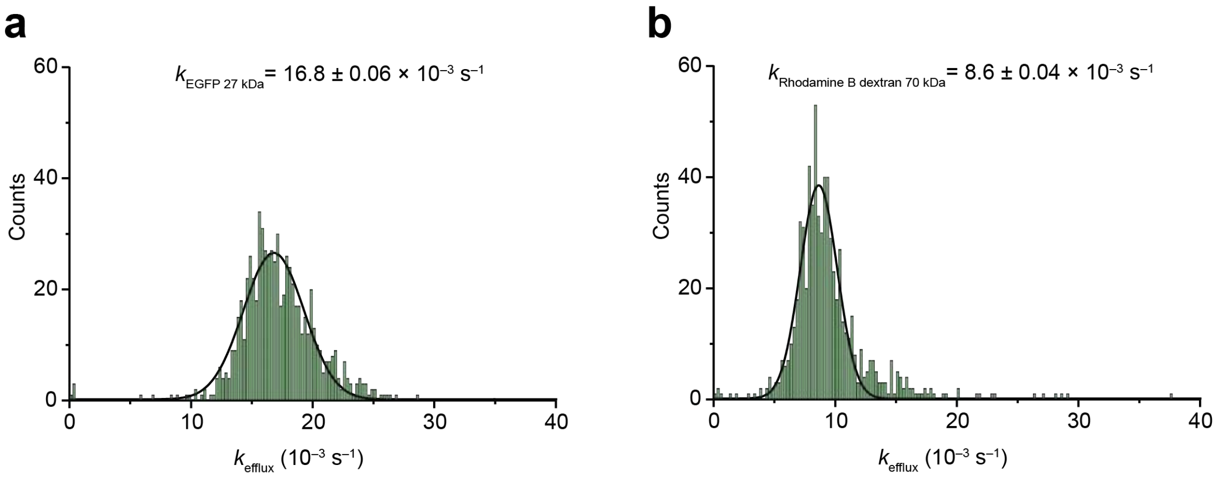

Supplementary Figure 15. Efflux constants after disruption of the supported lipid bilayer. Enhanced green fluorescent protein (EGFP) (5 µM, 27 kDa) and Rhodamine B dextran (8 µM, 70 kDa) were trapped inside the cavities. The supported lipid bilayer was solubilised by detergent Trion X-100 resulting in exponential efflux of both fluorescent analytes. The efflux traces were analysed to obtain rate constants. **a** Histogram displaying 703 rate constants for EGFP efflux after membrane solubilization. **b** Histogram of 698 rate constants for efflux of Rhodamine B dextran (70 kDa) after membrane solubilization. Source data are provided as a Source Data file.

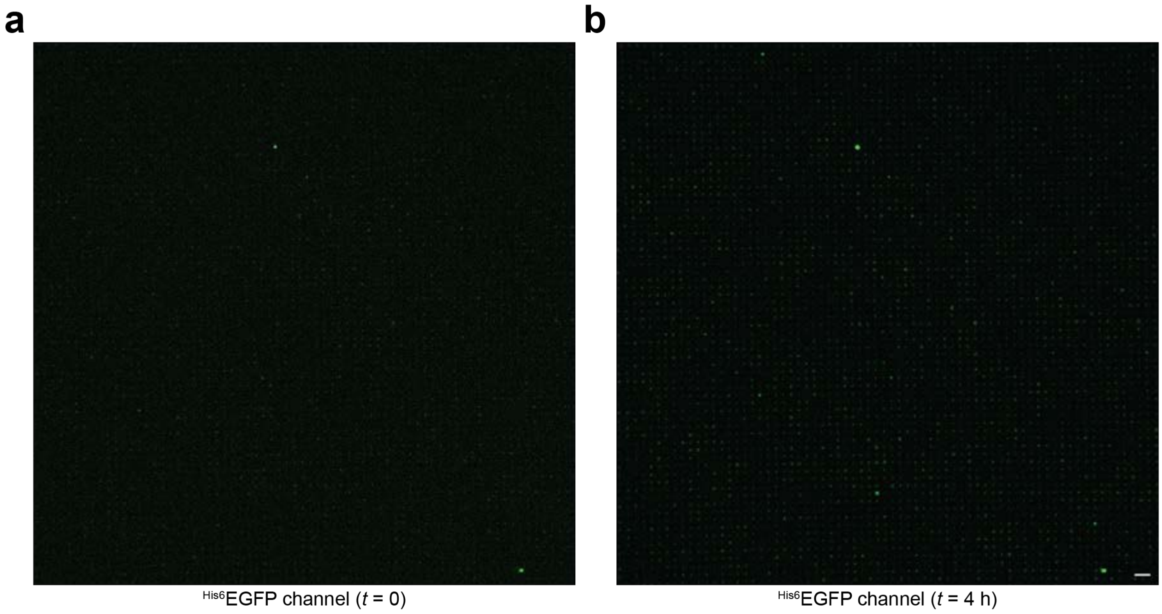

Supplementary Figure 16. Accumulation of green fluorescence in antibody sink experiments. Rhodamine B dextran (8 µM, 70 kDa) and antibody against His-tag (0.6 µM, 150 kDa) were encapsulated inside the cavities followed by liposome spreading. After supported lipid bilayer formation, hexahistidine-tagged enhanced green fluorescent protein (^{His6}EGFP) (80 nM, 27 kDa) was added to the buffer reservoir followed by addition of DNA nanopores. Upon pore-mediated flux into the cavities, ^{His6}EGFP was complexed by the His-tag antibody reducing the free ^{His6}EGFP concentration to allow further influx of the fluorescent protein. **a** ^{His6}EGFP fluorescence channel direct after the DNA nanopore addition. **b** ^{His6}EGFP fluorescence channel after 4 h of flux and DNA nanopore addition. Scale bar, 20 µm.

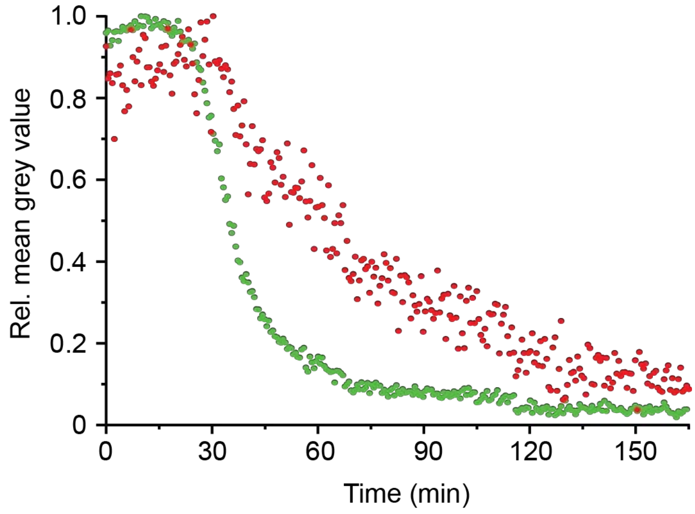

Supplementary Figure 17. Exemplary traces for the rupture of the supported lipid bilayer. Exemplary monoexponential efflux traces of hexahistidine-tagged enhanced green fluorescent protein (27 kDa, green circles) and Rhodamine B dextran (70 kDa, red circles) observed in the antibody control experiment. Source Data are provided as a Source Data file.

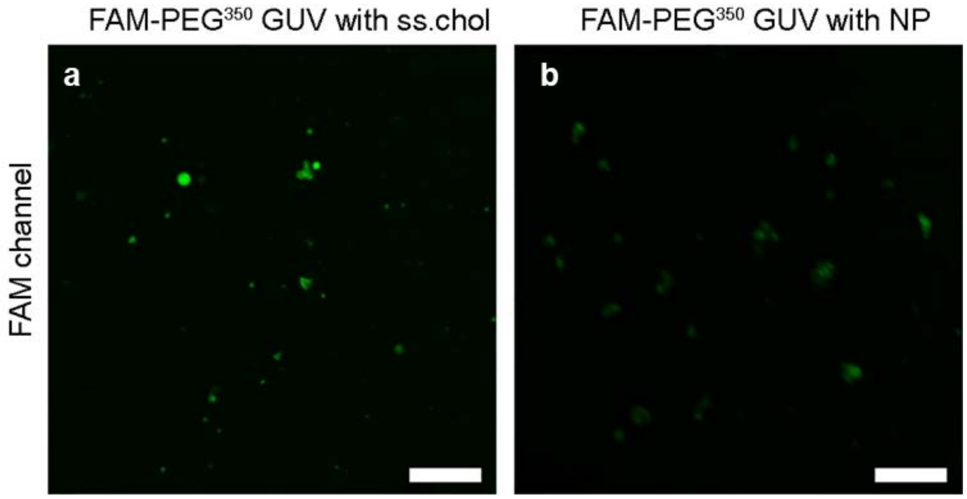

Supplementary Figure 18. NP Nanopore release fluorescent probe FAM-PEG^{350} from giant unilamellar vesicles. Fluorescence images of probe-filled vesicles incubated with cholesterol-modified DNA oligonucleotides (**a**) and NP (**b**)**.** The reduced fluorescence in the right image indicates that the pore transported molecular cargo across the membrane whereas cholesterol-oligonucleotides (left image) solely anchor to the membranes without puncturing it. Prior to microscopy analysis, GUVs were loaded with FAM fluorophore-tagged PEG^{350} of 2.4 nm hydrodynamic diameter[1, 2]. The size distribution of GUVs was within the norm[3].

Supplementary References

1. Merzlyak P. G., Yuldasheva L. N., Rodrigues C. G., Carneiro C. M. M., Krasilnikov O. V., Bezrukov S. M. Polymeric nonelectrolytes to probe pore geometry: Application to the alpha-toxin transmembrane channel. *Biophys. J.* **77**, 3023-3033 (1999).

2. Krasilnikov O. V., Rodrigues C. G., Bezrukov S. M. Single polymer molecules in a protein nanopore in the limit of a strong polymer-pore attraction. *Phys. Rev. Lett.* **97**, 018301-4 (2006).

3. Moscho A., Orwar O., Chiu D. T., Modi B. P., Zare R. N. Rapid preparation of giant unilamellar vesicles. *Proc. Natl. Acad. Sci. U S A* **93**, 11443-11447 (1996).
