# Supplementary material for: Synthetic protein-conductive membrane nanopores built with DNA
Source: Nat Commun. 2019 Nov 4;10:5018. doi: 10.1038/s41467-019-12639-y (PMC6828756; doi:10.1038/s41467-019-12639-y)
Supplement: Supplementary file 2 — Description of Additional Supplementary Files [file 41467_2019_12639_MOESM2_ESM.pdf]

Description of Additional Supplementary Files

File name: Supplementary Data 1

Description: Sequences of DNA staple, adaptor, and cholesterol-modified anchor oligonucleotides. All DNA sequences are from the 5' to the 3' terminus. The names of staple and adaptor oligonucleotides indicate their position within the 2D DNA map of the pore (Figure S2) described as helix and base pair coordinate for the strands' 3' terminus. The name of two cholesterol-modified anchor strands indicates whether the cholesterol-TEG modification is attached to the 5' or 3' terminus. Adaptor strands have two sequence parts which hybridize either to the DNA staple strands or the cholesterol-modified anchor oligonucleotides (highlighted in bold). Adaptors that carry the latter sequence part at the 5'-end hybridize to 3'-cholesterol anchor oligonucleotides. Conversely, adaptors with the sequence part at the 3'-terminus hybridize to 5'-cholesterol anchor oligonucleotides. Adapter strand 21[64] features both a 5'- and a 3'-terminal hybridization segment.
